# Effectiveness and safety of direct oral anticoagulants versus warfarin in patients with atrial fibrillation and advanced kidney disease

**DOI:** 10.1007/s11239-023-02859-x

**Published:** 2023-07-15

**Authors:** Chia-Chen Hsu, Cheng-Chi Chen, Chian-Ying Chou, Kuan-Hsuan Chen, Sheng-Fan Wang, Shih-Lin Chang, Yuh-Lih Chang

**Affiliations:** 1 Department of Pharmacy, Taipei Veterans General Hospital, No. 201, Sec. 2, Shih-Pai Road, Taipei 112, Taipei, Taiwan; 2 Department of Pharmacy, School of Pharmaceutical Sciences, National Yang Ming Chiao Tung University, Taipei, Taiwan; 3 Institute of Pharmacology, College of Medicine, National Yang Ming Chiao Tung University, Taipei, Taiwan; 4 Heart Rhythm Center, Division of Cardiology, Department of Medicine, Taipei Veterans General Hospital, Taipei, Taiwan; 5 Cardiovascular Research Center, School of Medicine, College of Medicine, National Yang Ming Chiao Tung University, Taipei, Taiwan

**Keywords:** Atrial fibrillation (AF), Chronic kidney disease (CKD), Direct oral anticoagulants (DOACs), Warfarin, Thrombosis, Hemorrhage

## Abstract

**Background:**

The effectiveness and safety of direct oral anticoagulants (DOACs) in patients with atrial fibrillation (AF) and advanced kidney disease (AKD) has not been fully established.

**Objectives:**

To determine the effectiveness and safety related to pooled or specific DOACs to that with warfarin in patients with AF and AKD.

**Methods:**

Patients with AF and AKD (estimated glomerular filtration rate < 30 mL/min) who received DOAC or warfarin from July 2011 to December 2020 were retrospectively identified in a medical center in Taiwan. Primary outcomes were hospitalized for stroke/systemic embolism and major bleeding. Secondary outcomes included any ischemia and any bleeding.

**Results:**

A total of 1,011 patients were recruited, of whom 809 (80.0%) were in the DOACs group (15.3% dabigatran, 25.4% rivaroxaban, 25.2% apixaban, and 14.1% edoxaban), and 202 (20.0%) in the warfarin group. DOACs had considerably lower risks of stroke/systemic embolism (adjusted hazard ratio [aHR] 0.29; 95% CI, 0.09–0.97) and any ischemia (aHR, 0.42; 95% CI, 0.22–0.79), but had comparable risks of major bleeding (aHR, 0.99; 95% CI, 0.34–2.92) and any bleeding (aHR, 0.74; 95% CI, 0.50–1.09) than warfarin. Apixaban was linked to considerably lower risks of any ischemia (aHR, 0.13; 95% CI, 0.04–0.48) and any bleeding (aHR, 0.53; 95% CI, 0.28–0.99) than warfarin.

**Conclusion:**

Among patients with AF and AKD, DOACs were linked to a lower risk of ischemic events, and apixaban was linked to a lower risk of any ischemia and any bleeding than warfarin.

**Supplementary Information:**

The online version contains supplementary material available at 10.1007/s11239-023-02859-x.

## Highlights

The effectiveness and safety of direct oral anticoagulants in patients with atrial fibrillation and advanced kidney disease has not been fully established.

DOACs were linked to lower risks of ischemic events compared with warfarin in patients with AF and AKD.

Apixaban was linked to a lower risk of any ischemia and any bleeding than warfarin in patients with AF and AKD.

**Supplementary Information:**

The online version contains supplementary material available at 10.1007/s11239-023-02859-x.

## Introduction

The global health incidence of atrial fibrillation (AF) and chronic kidney disease (CKD) is rising rapidly, and AF is highly prevalent in CKD patients (13–27%) and those on long-term dialysis (18%) [1–3]. Moreover, the presence of CKD is linked with an additional risk of thromboembolism and bleeding in patients with AF, and vice versa [4]. As a result, it is crucial to pursue the most adequate oral anticoagulant (OAC) to strike the balance between preventing ischemic stroke and mitigating bleeding events in AF patients with CKD.

Warfarin has been the mainstay of treatment in patients with AF and renal impairment for decades. However, warfarin has several limitations, including a narrow therapeutic window for safety, constant monitoring requirements, numerous diet, and drug-drug interactions [5]. Direct oral anticoagulants (DOACs) are relatively new agents, including dabigatran, rivaroxaban, apixaban, and edoxaban, which have been demonstrated to be superior or not inferior to warfarin in AF for efficiency and safety [6–10]. Furthermore, DOACs have fixed dosing regimens, which enhance the compliance and persistence with oral anticoagulant therapy. As a result, with the availability of DOACs, the prescription volumes of warfarin have decreased globally [11–15].

However, few randomized controlled trials of oral anticoagulants comprised patients with advanced kidney disease (AKD), estimated glomerular filtration rate (eGFR) < 30 mL/min. Although several regulatory agencies have authorized DOACs (except dabigatran) for patients with an eGFR above 15 mL/min on the basis of pharmacokinetic data, and a meta-analysis has validated the efficacy and safety of DOACs in this population, [16] the evidence of efficacy and safety between DOACs and warfarin remains low, particularly in comparisons between different DOACs. The disparities in efficacy and safety among DOACs in patients with AKD patients may be influenced by differences in their pharmacokinetic profiles. Existing studies mostly contrasted single DOAC (e.g., rivaroxaban, apixaban) [17–19] or pooled DOACs [20, 21] with warfarin, with less data on edoxaban or simultaneous comparison of the four DOACs individually with warfarin in AF patients with AKD. Therefore, this retrospective cohort study sought to assess the outcomes linked to pooled or specific DOACs compared with warfarin in patients with AF patients with AKD.

## Methods

### Data source & study design

We undertook a retrospective cohort study at Taipei Veterans General Hospital (TPEVGH), one of the largest medical centers in Taiwan, which allows more than 2.5 million outpatient visits for 1.1 million patients each year. This study was partially based on data from the Big Data Center, TPEVGH, and was authorized by the Institutional Review Board of TPEVGH (IRB-TPEVGH NO.: 2021-09-020BC). Informed consent was swayed due to the use of deidentified data and retrospective design.

Outpatients aged over 20 years and prescribed any oral anticoagulation between July 1, 2011, and December 31, 2020, were recruited. The cohort entry date was described as the date of initiation of treatment, and the date of OAC prescription plus eGFR < 30 mL/min constituted the index date. CrCl was measured according to the Cockcroft-Gault formula [22]. We exempted patients with (1) no visits or only one visit with a diagnosis of AF within 1 year before the OAC prescription; (2) eGFR ≥ 30 mL/min throughout the study period or unknown; (3) proof of anticoagulant prescription, knee/hip replacement surgery or venous thromboembolic events within 6 months before the cohort entry date (i.e., washout period); (4) history of valve surgery, mitral stenosis, or kidney transplant (see Table S1–S4 for codes).

Follow-up initiated the day after index date until the discovery of the outcomes, diversifying to other study drugs, discontinuation of anticoagulation prescription or > 30-day gap between new prescriptions, unknown/recovery of renal function (eGFR ≥ 30 mL/min) for over 6 months, withdrawal from valve surgery, kidney transplantation, or mitral stenosis, death, or study end (December 31, 2020), whichever came first (see Figure S1 for details).

### Ascertainment of exposure and outcome

All qualified participants were categorized into two groups: DOACs and the warfarin group. Warfarin was employed as an active comparator to enhance the study’s validity by mitigating measured and unmeasured differences between the study groups [23].

We analyzed hospitalization events using inpatient claims in the primary or secondary diagnosis position or relevant procedure codes (see Table S5–S7 for codes). First, two primary outcomes were admissions due to stroke/systemic embolism (stroke/SE) and major bleeding. Major bleeding was identified as hemorrhagic events that led to hospitalization. Second, the secondary effectiveness outcome was any ischemia, a composite of stroke/SE, venous thromboembolism, acute myocardial infarction, transient ischemic attack, and peripheral vascular disease. Finally, the secondary safety outcome was any bleeding, characterized as all bleeding events in inpatient and outpatient claims, whichever occurred first.

### Ascertainment of baseline covariates

We determined potential baseline confounders within one year before the index date. The covariates included demographic data, other medical comorbidities, and concomitant medications (see Table 1 for the list of covariates and Table S8–S12 for codes).

**Table 1:** Baseline characteristics of the study population before and after inverse probability of treatment weighting

	Before Weighting				After Weighting			
Variables	DOACs (N = 809)	Warfarin (N = 202)	Absolute standardized difference		DOACs (N = 503)	Warfarin (N = 508)	Absolute standardized difference	
**Demographics**								
Age, mean (SD), y	84.1 (7.4)	78.2 (10.3)	**0.66**		83.1 (6.6)	82.5 (12.6)	0.06	
Female sex, No. (%)	345 (42.7)	85 (42.1)	0.01		215 (42.7)	221 (43.6)	0.02	
Weight, mean (SD), kg	58.0 (11.1)	60.7 (12.1)	**0.23**		58.5 (8.8)	58.7 (17.5)	0.02	
eGFR, No. (%)			**0.82**				0.02	
15–29 mL/min	779 (96.3)	135 (66.8)			459 (91.3)	460 (90.7)		
< 15 mL/min, including dialysis^a^	30 (3.7)	67 (33.2)			44 (8.7)	48 (9.4)		
**Comorbidities, No. (%)**								
CHA_2_DS_2_-VASc score, mean (SD)	4.5 (1.4)	4.5 (1.7)	0.04		4.5 (1.1)	4.6 (2.3)	0.02	
HAS-BLED score, mean (SD)	3.3 (1.3)	3.6 (1.3)	**0.25**		3.3 (1.0)	3.4 (1.8)	0.02	
Quan-Charlson Comorbidity Index, mean (SD)	2.7 (2.2)	3.0 (2.1)	**0.14**		2.8 (1.8)	2.8 (3.5)	0.01	
Anemia	108 (13.4)	36 (17.8)	**0.12**		70 (13.9)	58 (11.5)	0.07	
Asthma	53 (6.6)	11 (5.5)	0.05		32 (6.3)	27 (5.4)	0.04	
Cancers	161 (19.9)	29 (14.4)	**0.15**		95 (19.0)	100 (19.8)	0.02	
Cerebrovascular disease	270 (33.4)	64 (31.7)	0.04		165 (32.8)	182 (35.8)	0.06	
Congestive heart failure	394 (48.7)	95 (47.0)	0.03		245 (48.8)	246 (48.6)	0.00	
Myocardial infarction	33 (4.1)	20 (9.9)	**0.23**		23 (4.7)	25 (5.0)	0.01	
Peripheral vascular disease	34 (4.2)	17 (8.4)	**0.17**		24 (4.9)	19 (3.7)	0.05	
Chronic obstructive pulmonary disorder	148 (18.3)	36 (17.8)	0.01		98 (19.5)	80 (15.8)	**0.10**	
Diabetes	273 (33.8)	87 (43.1)	**0.19**		182 (36.2)	200 (39.4)	0.07	
Gastrointestinal ulcer	134 (16.6)	34 (16.8)	0.01		85 (17.0)	66 (13.0)	**0.11**	
Hypertension	629 (77.8)	162 (80.2)	0.06		396 (78.7)	410 (80.8)	0.05	
Hyperlipidemia	215 (26.6)	69 (34.2)	**0.17**		144 (28.6)	152 (30.0)	0.03	
Liver disease	65 (8.0)	22 (10.9)	**0.10**		42 (8.3)	45 (8.9)	0.02	
Prior bleeding^b^	203 (25.1)	44 (21.8)	0.08		122 (24.3)	112 (22.1)	0.05	
Smoking			**0.13**				0.09	
Current non-smoker	756 (93.4)	191 (94.5)			474 (94.1)	484 (95.4)		
Current smoker	19 (2.4)	7 (3.5)			11 (2.2)	9 (1.8)		
Unknown	34 (4.2)	4 (2.0)			18 (3.7)	15 (2.8)		
Thyroid Disease	64 (7.9)	18 (8.9)	0.04		43 (8.5)	52 (10.3)	0.06	
Venous thromboembolism	16 (2.0)	3 (1.5)	0.04		10 (2.1)	18 (3.5)	0.09	
**Medication use, No. (%)**								
Antianxiety agents	220 (27.2)	65 (32.2)	**0.11**		143 (28.3)	131 (25.7)	0.06	
Antiarrhythmic agents	220 (27.2)	65 (32.2)	**0.11**		140 (27.8)	142 (28.1)	0.01	
Anti-depressants	92 (11.4)	21 (10.4)	0.03		60 (12.0)	68 (13.4)	0.04	
Antiplatelets	290 (35.9)	88 (43.6)	**0.16**		185 (36.8)	184 (36.3)	0.01	
Anti-hyperlipidemics	252 (31.2)	78 (38.6)	**0.16**		171 (33.9)	195 (38.5)	0.09	
ACEi / ARB	464 (57.4)	133 (65.8)	**0.18**		297 (59.1)	305 (60.0)	0.02	
β-Blockers	442 (54.6)	116 (57.4)	0.06		276 (54.8)	284 (56.0)	0.02	
Calcium channel blockers	546 (67.5)	142 (70.3)	0.06		336 (66.8)	351 (69.2)	0.05	
Diuretics	512 (63.3)	132 (65.4)	0.04		326 (64.7)	352 (69.3)	**0.10**	
Other anti-hypertensives	104 (12.9)	45 (22.3)	**0.25**		67 (13.3)	61 (12.0)	0.04	
Insulins	79 (9.8)	39 (19.3)	**0.27**		57 (11.4)	60 (11.8)	0.01	
Antidiabetics	200 (24.7)	64 (31.7)	**0.16**		132 (26.3)	157 (30.9)	**0.10**	
NSAIDs	198 (24.5)	40 (19.8)	**0.11**		122 (24.3)	133 (26.2)	0.04	
Proton pump inhibitors	140 (17.3)	48 (23.8)	**0.16**		93 (18.4)	99 (19.4)	0.03	

Abbreviations: ACEIs, angiotensin converting enzyme inhibitors; ARBs, angiotensin II receptor antagonists; DOACs, direct oral anticoagulants; eGFR, estimated glomerular filtration rate; NSAIDs, non-steroidal anti-Inflammatory drugs

^a^ Three (0.6%) patients in the DOACs group and 13 (2.6%) patients in the warfarin group received hemodialysis

^b^ Prior bleeding included gastrointestinal bleeding, intracranial hemorrhage, and other major bleeding, e.g., hematuria, epistaxis, and hemoptysiss

### Statistical analysis

We report the trends in oral anticoagulation prescriptions in the research population. For analysis, we applied the inverse probability of treatment weighting (IPTW) technique to balance the differences in baseline characteristics between treatment groups. All covariates were applied in a multivariate logistic regression model to forecast the probability of receiving DOACs versus warfarin. We weighted the patients by the inverse of this probability and stabilized the weights by multiplying by the number of patients in the treatment groups [24].

We utilized descriptive statistics in the study population before and after adopting IPTW, and the absolute standardized difference (ASD) ≥ 0.1 was interpreted as a potentially significant imbalance [25]. Survival free of an event in the weighted DOAC and warfarin cohorts was expressed with Kaplan-Meier curves and log-rank testing. Multivariate Cox proportional hazard regression models weighted with IPTW were employed to calculate adjusted hazard ratios (aHRs) and 95% CIs. Variables that were significant (ASD ≥ 0.1) and clinically relevant confounders (i.e., age, sex, CHA_2_DS_2_-VASc/HAS-BLED score, smoking status, prior bleeding, cerebrovascular disease, myocardial infarction, peripheral vascular disease, venous thromboembolism, antiplatelets, non-steroidal anti-inflammatory drugs) were included in the multivariate model. For the sub-analysis, we segmented the DOACs group in the main analysis into four cohorts: the dabigatran, rivaroxaban, apixaban, and edoxaban cohorts, and compared with warfarin, respectively.

### Sensitivity and subgroup analyses

A series of sensitivity analysis was conducted to validate the results. Specifically, we (1) excluded unreasonable doses of DOACs; (2) only included warfarin users with time in the therapeutic range (TTR) ≥ 70% of their international normalized ratio (INR) readings between 1.5 and 3.0;[26–29]. (3) utilized the Modification of Diet in Renal Disease (MDRD) Study equation to calculate GFR;[30]. (4) restricted the follow-up period to only 1 year; (5) removed patients with eGFR < 30 mL/min only once.

Furthermore, subgroup analysis was conducted to address the potential prevalent user bias. To maximize our sample size, we included the prevalent users in this research. Thus, new users were regarded as the initiation of treatment at eGFR < 30 mL/min, whereas prevalent users started treatment at eGFR ≧ 30 mL/min. We performed a subgroup analysis to reveal whether the connection between these groups was coherent. We employed Cochran’s Q heterogeneity statistic in the sensitivity and subgroup analysis to detect interactions.

All data management and analysis were carried out using Statistical Analysis System, version 9.4 (SAS Institute, Cary, NC, USA) and R Statistical Software version 4.1.3 (Foundation for Statistical Computing). Two-sided *P* < 0.05 were deemed statistically significant.

## Results

We recruited 1,011 patients with AF and eGFR less than 30 mL/min. Among them, 809 (80.0%) were in the DOACs group, with 19.2% (n = 155) prescribed dabigatran, 31.8% (n = 257) prescribed rivaroxaban, 31.5% (n = 255) prescribed apixaban, 17.6% (n = 142) prescribed edoxaban (see Table S13 for details). The remaining 202 patients (20.0%) were in the warfarin group, with a mean TTR of 39.5% (see Table S14 for TTR details). The flow chart for the enrollment of the study population is depicted in Figure S2.

### Trends of OAC prescription

**Fig. 1:**
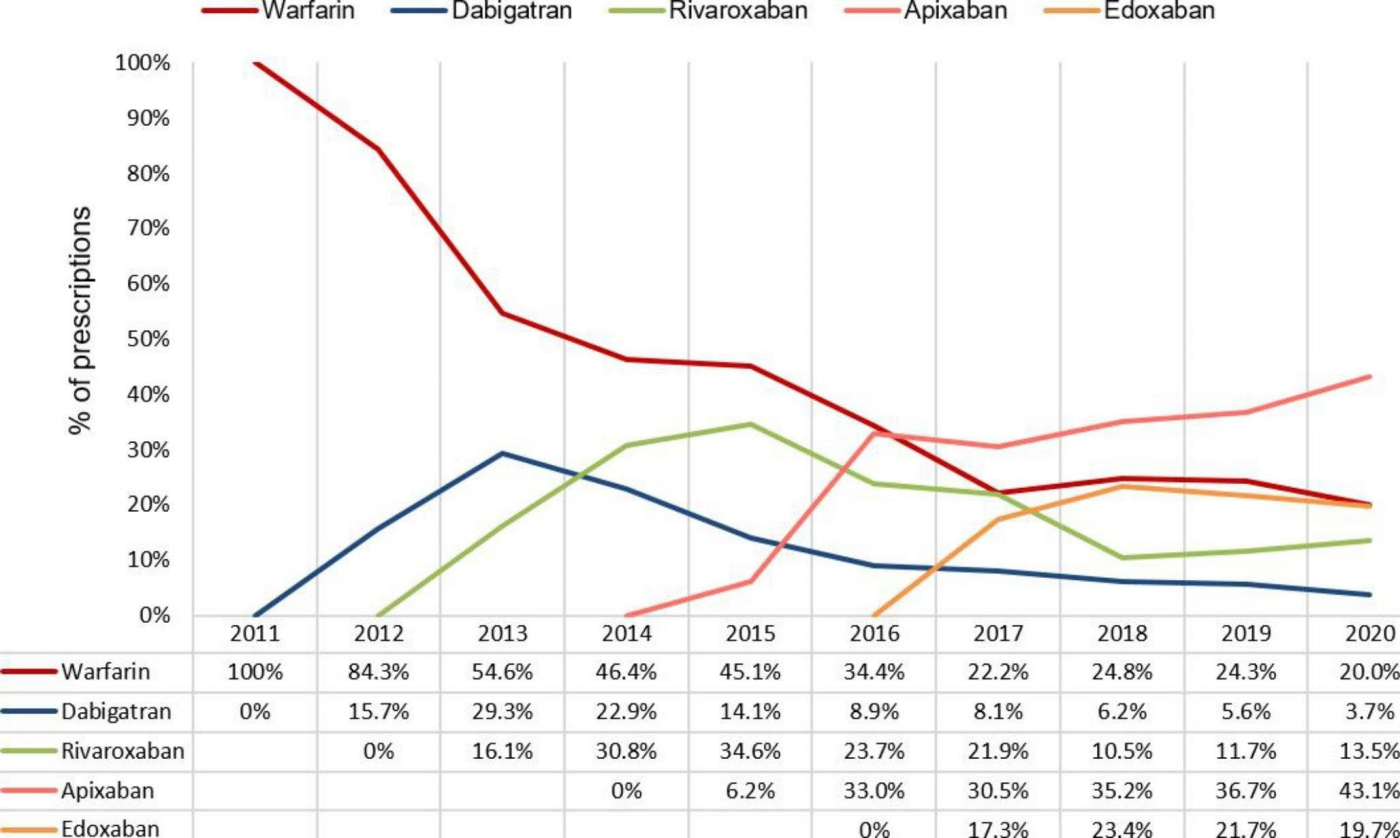
Changes in oral anticoagulant prescriptions among patients with advanced kidney disease and atrial fibrillation (AF) throughout the research (2011–2020)

The trends in OAC prescription in the study population are depicted in Fig. 1. Dabigatran, rivaroxaban, apixaban, and edoxaban were previously prescribed in 2012, 2013, 2015, and 2017, respectively. After the development of DOACs, the percentage of warfarin decreased markedly over the period (100% in 2011 and 20% in 2020). Similarly, a downward tendency can be observed in dabigatran and rivaroxaban, after a rapid elevation to a peak of 29.3% in 2013 and 34.6% in 2015, respectively. In contrast, the percentage of apixaban gradually increased and apixaban use (30.5%) exceeded warfarin use (22.5%) in 2017. By 2020, apixaban use was still prevalent (43.1%). The percentage of edoxaban was constant at around 20% between 2017 and 2020.

### Baseline patient characteristics

Prior to implementing IPTW, DOACs users were older (84.1 versus 78.2 years), had lower body weight (58.0 versus 60.7 kg), a lower proportion of patients with eGFR < 15 mL/min (3.7 versus 33.2%), a lower HAS-BLED score (3.3 versus 3.6), and a lower Quan-Charlson Comorbidity Index (2.7 versus 3.0) compared to warfarin users. The mean scores for CHA_2_DS_2_-VASc and HAS-BLED were 4.5 ± 1.4, 4.5 ± 1.7 and 3.3 ± 1.3, 3.6 ± 1.3, respectively, validating that these patients tend to thrombosis and hemorrhage. IPTW successfully achieved balance in all baseline characteristics, except for COPD, GI ulcer, use of diuretics, and use of antidiabetics.

### Clinical outcomes

**Fig. 2:**
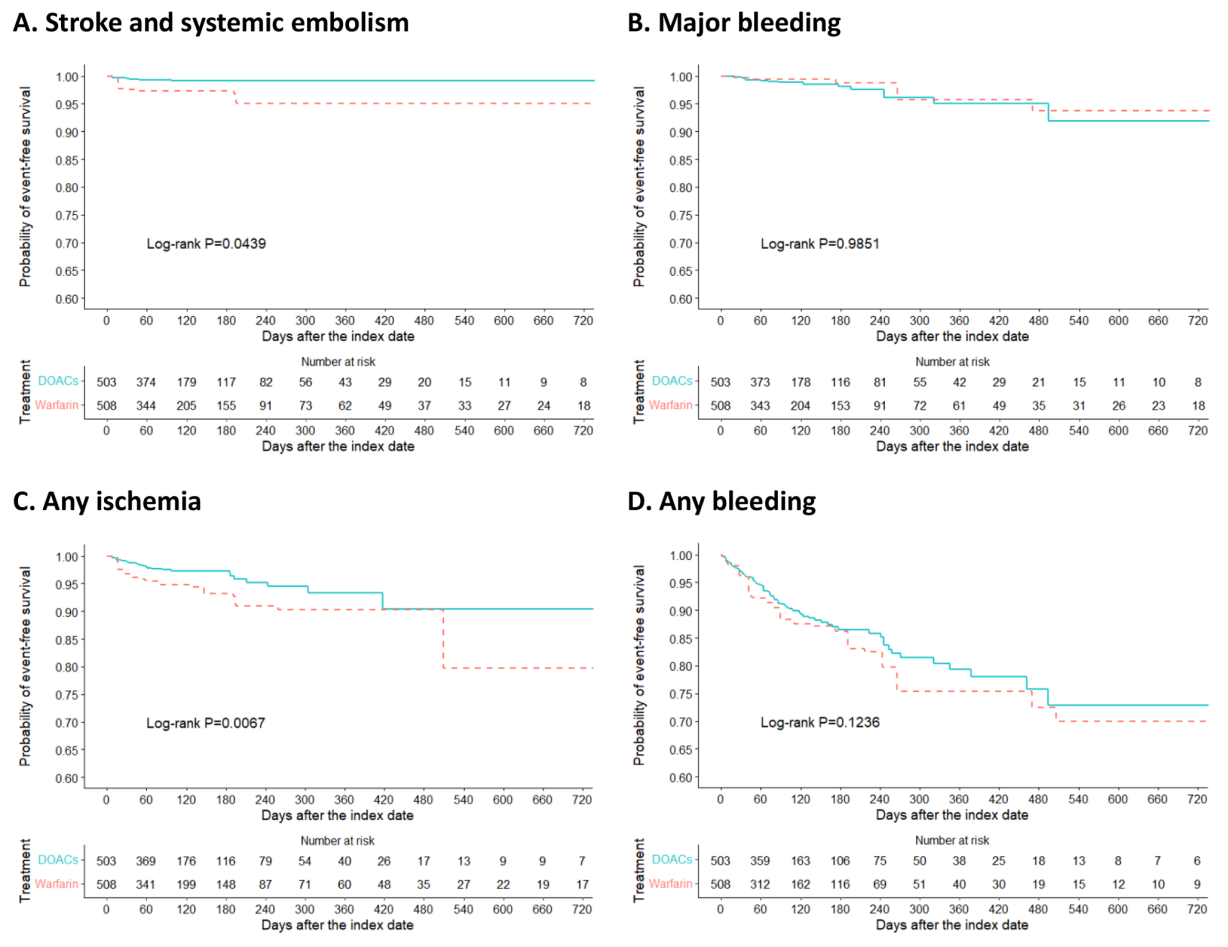
Inverse probability of treatment weighting (IPTW) Kaplan-Meier survival curves in patients with advanced kidney disease and atrial fibrillation (AF). **(A)** Stroke/systemic embolism. **(B)** Major bleeding. **(C)** Any ischemia. **(D)** Any bleeding. DOACs indicates direct oral anticoagulants

The incidence rates and aHRs of outcomes are expressed in Table 2, and Kaplan-Meier survival curves of outcomes after integrating IPTW are depicted in Fig. 2 (see Table S15 and Figure S3 for unweighted results). The incidence rate of stroke/SE was 2.25 and 6.54 per 100 patient-years for the DOACs and warfarin groups with a considerably lower risk of stroke/SE between the groups (log-rank *P =* 0.0439). In multivariate Cox regression analysis after IPTW, the aHR for DOACs versus warfarin was 0.29 (95% CI, 0.09–0.97; *P* = 0.0439) for stroke/SE. No substantial difference between the two groups was found for major bleeding (4.33 and 2.74 per 100 patient-years for DOACs and warfarin, respectively) with a non-significant association estimate (aHR, 0.99; 95% CI, 0.34–2.92; *P* = 0.9851). Furthermore, DOACs were linked to a significantly lower risk of any ischemia (aHR, 0.42; 95% CI, 0.22–0.79; *P* = 0.0067). Finally, there was a non-significant trend toward less bleeding in the DOACs group (aHR, 0.74; 95% CI, 0.50–1.09; *P =* 0.1236). The reasons for the censorship are presented in Table S16.

**Table 2:** Incidence rates and hazard ratios of outcomes after inverse probability of treatment weighting

Outcome	DOACs group (n = 503)				Warfarin group (n = 508)			Adjusted HR (95% CI)^b^
	Events	PY	Rate (95%CI)^**a**^		Events	PY	Rate (95%CI)^**a**^	
Stroke/SE	5	208	2.25 (0.91–5.56)		17	259	6.54 (4.06–10.53)	0.29 (0.09–0.97)
Major bleeding	9	208	4.33 (2.25–8.33)		7	257	2.74 (1.31–5.74)	0.99 (0.34–2.92)
Any ischemia	17	202	8.27 (5.12–13.34)		35	248	14.18 (10.18–19.74)	0.42 (0.22–0.79)
Any bleeding	55	194	28.38 (21.80-36.95)		65	202	32.31 (25.33–41.20)	0.74 (0.50–1.09)

^a^ Incidence rate, per 100 person-years

^b^ Weighted with inverse probability of treatment weighting (IPTW) and adjusted for age, sex, chronic obstructive pulmonary disease, gastrointestinal ulcer, diuretics, antidiabetics, CHA_2_DS_2_-VASc score, HAS-BLED score, smoking status, prior bleeding, cerebrovascular disease, myocardial infarction, peripheral vascular disease, venous thromboembolism, antiplatelets, non-steroidal anti-inflammatory drugs

Abbreviations: DOACs, direct oral anticoagulants; PY person-years; SE systemic embolism

**Fig. 3:**
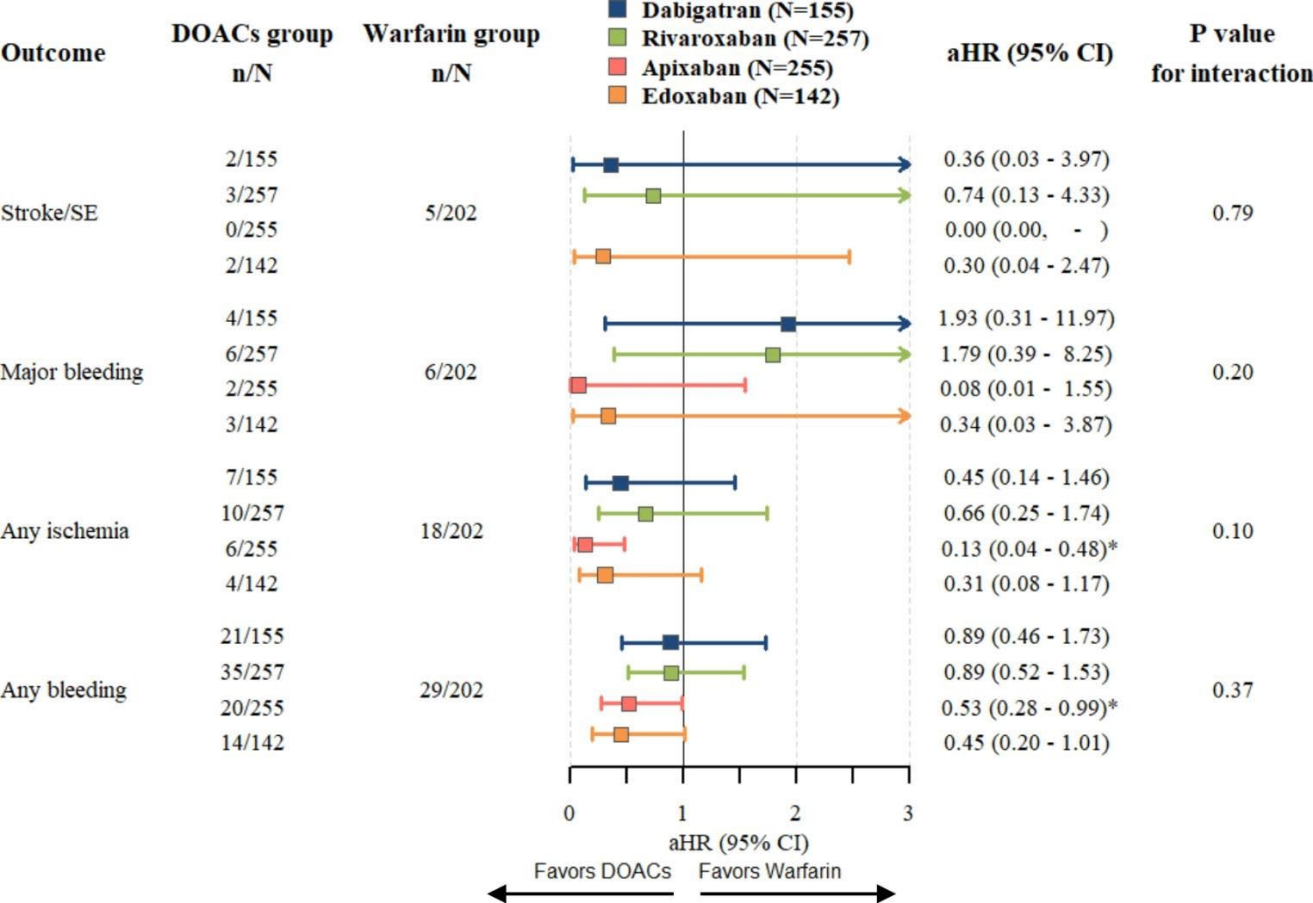
DOAC-specific sub-analysis compared with warfarin and P value for interaction. Hazard ratios and 95% CIs are derived from Cox regression analyses weighted with IPTW and adjusted for age, sex, comorbidities, and comedications. DOACs indicates direct oral anticoagulants; aHR, adjusted hazard ratio; CI, confidence interval; n, number in group with the outcome; and N, total number in group

### DOAC-specific analyses

The sub-analysis (Fig. 3) demonstrated that each specific DOAC may be linked to lower risks in stroke/SE, any ischemia, and any bleeding. Each DOAC (except apixaban, as no event was observed and HR could not be calculated) indicated a comparable but not significant trend in stroke/SE. The risks of major bleeding were inconsistent with each DOAC. While dabigatran (aHR, 1.93; 95% CI, 0.31–11.97; *P* = 0.4806) and rivaroxaban (aHR, 1.79; 95% CI, 0.39–8.25; *P* = 0.4546) may be linked to a higher risk of major bleeding, apixaban (aHR, 0.08; 95% CI, 0.01–1.55; *P* = 0.0942) and edoxaban (aHR, 0.34; 95% CI, 0.03–3.87; *P* = 0.3818) may lower the risks of major bleeding, compared with warfarin. Apixaban was related to a significant reduction in the risk of any ischemia (aHR, 0.13; 95% CI, 0.04–0.48; *P* = 0.0021) and any bleeding (aHR, 0.53; 95% CI, 0.28–0.99; *P* = 0.0470) than warfarin. Edoxaban also exhibited a comparable but not significant trend (aHR, 0.31; 95% CI, 0.08–1.17; *P* = 0.0831 in any ischemia; aHR, 0.45; 95% CI, 0.20–1.01 in any bleeding).

### Sensitivity and subgroup analysis

We did not discover any significance in the sensitivity and subgroup analysis (see Table S17 and Fig. 4 for details), except for any bleeding in the sensitivity analysis of only including warfarin users with TTR ≥ 70% (HR, 1.89; 95% CI, 0.69–5.19; *P* for interaction *=* 0.09).

## Discussion

In this retrospective cohort study, we discovered that the use of DOACs elevated with a corresponding decline in warfarin in patients with AF and AKD. DOACs substantially decreased the risk of stroke/SE and any ischemia in patients with AF and AKD compared with warfarin. In the sub-analysis of each DOAC, apixaban was linked to a significant reduction in the risk of any ischemia and any bleeding compared with warfarin.

### Trends of OAC prescription

In the current study, we observed that the percentage of DOAC use in patients with AF and AKD has consistently increased in the last decade, with a corresponding decline in warfarin. A similar trend was found in the other studies of AF patients with chronic renal disease [12, 31–34]. We discovered that rivaroxaban and apixaban were the two most prevalently prescribed DOACs, which is coherent with prior studies in AF patients [35, 36]. The increasing use of DOACs highlights the importance of their use in AKD populations to assess efficacy and safety, necessitating the need for additional evidence.

### Clinical outcomes

Based on our findings, DOACs seem to be more efficient than warfarin in preventing ischemic stroke/systemic embolism and any ischemia events among patients with AF patients with AKD. A multicentre retrospective cohort study, also undertaken in Taiwan, revealed similar results [20]. A systematic review and meta-analysismerging data from various observational studies of this population discovered similar outcomes. [16].

In the present study, we observed a significant disparity in the distribution of individuals with end-stage renal disease (ESRD, eGFR < 15 mL/min with or without dialysis) between DOACs and warfarin (DOACs 3.7% versus warfarin 33.2%). This finding aligns with the results reported by Betra et al., indicating that warfarin remains the preferred OAC choice for patients with ESRD [31]. Previous meta-analyses comparing DOACs with warfarin in the ESRD population, primarily focusing on dialysis patients, have yielded inconsistent outcomes. See et al. reported no significant difference in effectiveness and safety outcomes between DOACs and warfarin in AF patients on dialysis [37]. In contrast, Elfar et al. demonstrated that DOACs were associated with higher rates of systemic embolization, minor bleeding, and death compared to warfarin [38]. Conversely, Li et al. found that DOACs were associated with a reduced risk of gastrointestinal bleeding [39]. Furthermore, none of the studies have specifically examined ESRD patients without dialysis. Therefore, further studies are needed to validate OAC selection for the AF patients with ESRD.

A previous study demonstrated a progressive increase in the incidence of net adverse clinical events (including death, ischemic stroke, systemic embolism, and major bleeding) as renal function deteriorated [40]. The benefit of anticoagulation therapy varies among patients with different stages of CKD. In our study, the primary population consisted of individuals in CKD stage 4, and the findings may not be generalizable to stage 5. Furthermore, the disparities in the percentage of each DOAC item between studies have restricted the interpretation and applicability of these results. Therefore, direct head-to-head comparisons using individual-level data are required to fully determine the comparative effects of DOACs.

### DOAC-specific analyses

In the DOAC-specific analyses, we observed that apixaban was the only DOAC that markedly reduced the risks of any ischemia and bleeding compared with warfarin. Prior studies have also supplemented the application of apixaban in AKD patients to mitigate the risk of major bleeding and have the potential to lower the risk of ischemic events [18, 33, 41]. The varying proportion of renal elimination could partially explain the irregularity in embolic and bleeding outcomes among DOACs. Apixaban is less dependent on renal elimination than the other DOACs, [42] and is the only DOAC authorized for application in hemodialysis based on pharmacokinetic data [43].

The available evidence on the use of edoxaban in patients with advanced kidney disease is limited. A 12-week phase 3 study conducted in Japanese AF patients with an eGFR of 15–29 mL/min showed that a once-daily dose of 15 mg of edoxaban had a safety and pharmacokinetic profile similar to the 30 and 60 mg doses in patients with normal renal function [44]. Additionally, a small retrospective study reported that the use of edoxaban in AF patients with an eGFR of 15–29 mL/min were not observed major bleeding or thrombotic events [45]. Our findings indicate that edoxaban may be linked to a decreased risk of embolic and bleeding events. Although our results did not reach statistical significance, there is a trend suggesting a lower risk of both embolic and bleeding events compared to warfarin.

These findings from DOAC-specific analyses support the hypothesis that the bleeding and ischemic risks associated with DOACs may differ in patients with advanced kidney disease, highlighting the potential for personalized drug selection in real-world scenarios.

### Study strengths and limitations

The current study has multiple main strengths. First, we applied comprehensive laboratory data (e.g., serum creatinine, weight, height, etc.) instead of diagnostic codes to specifically capture our study population. Second, we highlighted the effectiveness and safety of the four DOACs separately in this special population. Finally, we examined the effect of different scenarios in the sensitivity analyses and dealt with the prevalent user bias properly in the subgroup analysis to evaluate our study outcomes.

Despite these strengths, this research has the following limitations. First, as the retrospective observational study design, we could not access all residual confounders (e.g., drug adherence), although we controlled for age, sex, comorbidities, medications, CHA2DS2-VASc score, HAS-BLED score, and smoking status in Cox regression models. Second, since our data are obtained from a single center, we may unavoidably underestimate dialysis recipients and lethal outcomes, and also have lower external validity to other ethnic groups. Third, the primary population consisted of individuals in CKD stage 4, and the findings may not be generalizable to stage 5. Finally, given the limitations in sample size, there may have been insufficient statistical power to compare certain individual direct oral anticoagulants (DOACs) with warfarin. However, these findings have the potential to support the feasibility of personalized drug selection in real-world settings.

In conclusion, the application of DOACs was linked to lower risks of ischemic events compared with warfarin in patients with AF and AKD. Among DOACs, apixaban was connected to a substantial reduction in the risk of any ischemia and any bleeding compared with warfarin.

### Electronic supplementary material

Below is the link to the electronic supplementary material.

Supplementary Material 1

## Declarations

**of Conflicting Interests**.
