# Supplementary material for: Effectiveness and safety of direct oral anticoagulants versus warfarin in patients with atrial fibrillation and advanced kidney disease
Source: J Thromb Thrombolysis. 2023 Jul 15;56(4):518–28. doi: 10.1007/s11239-023-02859-x (PMC10550875; doi:10.1007/s11239-023-02859-x)
Supplement: Supplementary file 1 — Supplementary Material 1 [file 11239_2023_2859_MOESM1_ESM.docx]

# Effectiveness and Safety of Direct Oral Anticoagulants versus Warfarin in Patients with Atrial Fibrillation and Advanced Kidney Disease

# Table S1. Codes for oral anticoagulants

| **Generic name** | **ATC code** | **NHI code** | | |
| --- | --- | --- | --- | --- |
| Dabigatran | B01AE07 | B025458100  B025459100 | BC25458100  BC25459100 | BC26233100 |
| Rivaroxaban | B01AF01 | AC60205100  B025129100  B025647100  BC27734100 | BC25129100  BC25647100  BC25648100  BC27733100 | B025648100  BC27735100  BC27750100  XC00195100 |
| Apixaban | B01AF02 | BC26124100  BC26133100 | BC27783100 | BC27789100 |
| Edoxaban | B01AF03 | BC26599100 | BC26600100 | BC26601100 |
| Warfarin | B01AA03 | A043862100  A050095100  A050423100  A052559100  AC43862100  AC50095100  AC50423100  AC52559100 | AC55271100  AC60168100  AC60318100  AC60391100  AC60563100  B020346100  B020354100  B020515100 | B020516100  B023426100  B023572100  B023573100  BC23572100  BC23573100  X000140100 |

**Abbreviations:** ATC, Anatomical Therapeutic Chemical; NHI, National Health Insurance

# Table S2. Codes for identification of atrial fibrillation

| **Condition** | **ICD-9-CM diagnosis code** | **ICD-10-CM diagnosis code** |
| --- | --- | --- |
| Atrial fibrillation | 427.3x | I48.x |

**Abbreviations:** ICD-9-CM, International Classification of Diseases, Ninth Revision, Clinical Modification; ICD-10-CM, International Classification of Diseases, Tenth Revision, Clinical Modification

# Table S3. Codes used to exclude patients who had knee/hip replacement surgery or thromboembolic events within 6 months prior to the cohort entry date

Table S3.1. Venous thromboembolism diagnosis codes

| **Condition** | **ICD-9-CM diagnosis code** | **ICD-10-CM diagnosis code** |
| --- | --- | --- |
| Venous thromboembolism | 415.1x, 451.x, 452.x, 453.x | I26.9x, I80.x, I81.x, I82.x |

Table S3.2. Hip/knee replacement surgery procedure codes

| **Procedure** | **Code type** | **Codes** |
| --- | --- | --- |
| Hip or Knee arthroplasty (primary or revision) | ICD-9-CM Procedure | 00.70, 00.71, 00.72, 00.73, 00.80, 00.81, 00.82, 00.85, 00.86, 00.87, 81.51, 81.52, 81.53, 81.54 |
|  | ICD-10-PCS Procedure | See table below. |

Table S3.3. ICD-10-PCS codes for hip/knee replacement surgery (primary or revision)

| **Procedure** | **ICD-10-PCS procedure code** | | | | |
| --- | --- | --- | --- | --- | --- |
| Hip arthroplasty (primary or revision) | 0SPA0JZ  0SPA4JZ  0SPE0JZ  0SPE4JZ  0SPR0JZ  0SPR4JZ  0SPS0JZ  0SPS4JZ  0SR9019  0SR901A  0SR901Z  0SR9029  0SR902A  0SR902Z  0SR9039  0SR903A  0SR903Z  0SR9049  0SR904A  0SR904Z  0SR9069  0SR906A  0SR906Z  0SR907Z  0SR90J9 | 0SR90JA  0SR90JZ  0SR90KZ  0SRA009  0SRA00A  0SRA00Z  0SRA019  0SRA01A  0SRA01Z  0SRA039  0SRA03A  0SRA03Z  0SRA07Z  0SRA0J9  0SRA0JA  0SRA0JZ  0SRA0KZ  0SRB019  0SRB01A  0SRB01Z  0SRB029  0SRB02A  0SRB02Z  0SRB039  0SRB03A | 0SRB03Z  0SRB049  0SRB04A  0SRB04Z  0SRB069  0SRB06A  0SRB06Z  0SRB07Z  0SRB0J9  0SRB0JA  0SRB0JZ  0SRB0KZ  0SRE009  0SRE00A  0SRE00Z  0SRE019  0SRE01A  0SRE01Z  0SRE039  0SRE03A  0SRE03Z  0SRE07Z  0SRE0J9  0SRE0JA | 0SRE0JZ  0SRE0KZ  0SRR019  0SRR01A  0SRR01Z  0SRR039  0SRR03A  0SRR03Z  0SRR07Z  0SRR0J9  0SRR0JA  0SRR0JZ  0SRR0KZ  0SRS019  0SRS01A  0SRS01Z  0SRS039  0SRS03A  0SRS03Z  0SRS07Z  0SRS0J9  0SRS0JA  0SRS0JZ  0SRS0KZ | 0SU90BZ  0SUA0BZ  0SUB0BZ  0SUE0BZ  0SUR0BZ  0SUS0BZ  0SW90JZ  0SW93JZ  0SW94JZ  0SWA0JZ  0SWA3JZ  0SWA4JZ  0SWB0JZ  0SWB3JZ  0SWB4JZ  0SWE0JZ  0SWE3JZ  0SWE4JZ  0SWR0JZ  0SWR3JZ  0SWR4JZ  0SWS0JZ  0SWS3JZ  0SWS4JZ |
| Knee arthroplasty (primary or revision) | 0SPC0JC  0SPC4JC  0SPD0JC  0SPD4JC  0SPT0JZ  0SPT4JZ  0SPU0JZ  0SPU4JZ  0SPV0JZ  0SPV4JZ  0SPW0JZ  0SPW4JZ  0SRC069  0SRC06A  0SRC06Z  0SRC07Z | 0SRC0J9  0SRC0JA  0SRC0JZ  0SRC0KZ  0SRC0L9  0SRC0LA  0SRC0LZ  0SRD069  0SRD06A  0SRD06Z  0SRD07Z  0SRD0J9  0SRD0JA  0SRD0JZ  0SRD0KZ  0SRD0L | 0SRD0LA  0SRD0LZ  0SRT07Z  0SRT0J9  0SRT0JA  0SRT0JZ  0SRT0KZ  0SRU07Z  0SRU0J9  0SRU0JA  0SRU0JZ  0SRU0KZ  0SRV07Z  0SRV0J9  0SRV0JA  0SRV0JZ | 0SRV0KZ  0SRW07Z  0SRW0J9  0SRW0JA  0SRW0JZ  0SRW0KZ  0SWC0JC  0SWC0JZ  0SWC3JC  0SWC3JZ  0SWC4JC  0SWC4JZ  0SWD0JC  0SWD0JZ  0SWD3JC | 0SWD3JZ  0SWD4JC  0SWD4JZ  0SWT0JZ  0SWT3JZ  0SWT4JZ  0SWU0JZ  0SWU3JZ  0SWU4JZ  0SWV0JZ  0SWV3JZ  0SWV4JZ  0SWW0JZ  0SWW3JZ  0SWW4JZ |

**Abbreviations:** ICD-9-CM, International Classification of Diseases, Ninth Revision, Clinical Modification; ICD-10-PCS, International Classification of Diseases, Tenth Revision, Procedure Coding System

# Table S4. Codes used to exclude patients with the history of valve surgery, mitral stenosis, or kidney transplant

Table S4.1. Codes for medical conditions

| **Condition** | **ICD-9-CM diagnosis code** | **ICD-10-CM diagnosis code** |
| --- | --- | --- |
| Mitral stenosis | 394.0, 394.2, 396.0, 396.1, 396.8, 396.9, 424.0, 746.5 | I05.0, I05.2, I08.0, I08.1, I08.3, I08.8, I08.9, I34, I34.2, Q23.2, Z95.2, Z95.3 |
| Heart valve replacement | V42.2, V43.3, 996.02, 996.71 | Z95.4, T82.0x, T82.22x, T82.827x, T82.867x, T82.897x, T82.9x, T82.837x, T82.847x, T82.857x |
| Renal transplant | V42.0 | Z94.0 |

Table S4.2. Codes for medical procedures

| **Procedure** | **Code type** | **Codes** | | | | |
| --- | --- | --- | --- | --- | --- | --- |
| Valve surgery | ICD-9-CM Procedure | 35.00, 35.01, 35.02, 35.03, 35.04, 35.10, 35.11, 35.12, 35.13, 35.14, 35.20, 35.21, 35.22, 35.23, 35.24, 35.25, 35.26, 35.27, 35.28, 35.96, 35.99, 35.0x, 35.1x, 35.2x | | | | |
|  | ICD-10-PCS Procedure | 024F07J  024G082  024J0K2  027H0ZZ  02NF0ZZ  02QF0ZZ  02QG3ZE  02QJ0ZG  02QJ4ZZ  02RF37H  02RF3JZ  02RG0JZ  02RG4JZ  02RH37Z  02RH3KH  02RJ0JZ  02UF07Z  02UF37J  02UF3JZ  02UF4KZ  02UG0KE  02UG3KZ  02UH3JZ  02UJ3JG  02WF3JZ  02WH0JZ  02WJ4JZ | 024F08J  024G0J2  027F0ZZ  027H3ZZ  02NG0ZZ  02QF3ZZ  02QG4ZE  02QJ0ZZ  02RF07Z  02RF37Z  02RF3KZ  02RG0KZ  02RH07Z  02RH38H  02RH3KZ  02RJ0KZ  02UF0JJ  02UF37Z  02UF47Z  02UG07E  02UG37Z  02UG4JE  02UH4JZ  02UJ3JZ  02WF4JZ  02WH3JZ  X2RF032 | 024F0JJ  024J072  027F3ZZ  027J0ZZ  02NH0ZZ  02QF4ZZ  02QH0ZZ  02QJ3ZG  02RF08Z  02RF38H  02RF4JZ  02RG3JH  02RH08Z  02RH38Z  02RH4JZ  02RJ3JH  02UF0JZ  02UF38J  02UF4JJ  02UG08E  02UG3JE  02UG4JZ  02UJ0JG  02UJ4JG  02WG0JZ  02WH4JZ  X2RF332 | 024F0KJ  024J082  027G0ZZ  027J3ZZ  02NJ0ZZ  02QG0ZE  02QH3ZZ  02QJ3ZZ  02RF0JZ  02RF38Z  02RG07Z  02RG3JZ  02RH0JZ  02RH3JH  02RJ07Z  02RJ3JZ  02UF0KJ  02UF38Z  02UF4JZ  02UG0JE  02UG3JZ  02UH0JZ  02UJ0JZ  02UJ4JZ  02WG3JZ  02WJ0JZ  X2RF432 | 024G072  024J0J2  027G3ZZ  02BK0ZZ  02QF0ZJ  02QG0ZZ  02QH4ZZ  02QJ4ZG  02RF0KZ  02RF3JH  02RG08Z  02RG3KH  02RH0KZ  02RH3JZ  02RJ08Z  02RJ4JZ  02UF0KZ  02UF3JJ  02UF4KJ  02UG0JZ  02UG3KE  02UH37Z  02UJ0KG  02WF0JZ  02WG4JZ  02WJ3JZ |

**Abbreviations:** ICD-9-CM, International Classification of Diseases, Ninth Revision, Clinical Modification; ICD-10-CM, International Classification of Diseases, Tenth Revision, Clinical Modification

# Table S5. Codes for ischemic stroke/systemic embolism events

| **Outcome Subtype** | **ICD-9-CM diagnosis code** | **ICD-10-CM diagnosis code** |
| --- | --- | --- |
| Ischemic stroke | 433.01, 433.11, 433.21, 433.31, 433.81, 433.91, 434.01, 434.11, 434.91, 436.x | I63 |
| Systemic embolism | 444 | I74 |

**Abbreviations:** ICD-9-CM, International Classification of Diseases, Ninth Revision, Clinical Modification; ICD-10-CM, International Classification of Diseases, Tenth Revision, Clinical Modification

# Table S6. Codes for any ischemic event

Table S6.1. Codes for medical conditions

| **Outcome Subtype** | **ICD-9-CM diagnosis code** | **ICD-10-CM diagnosis code** |
| --- | --- | --- |
| Ischemic stroke | 433.01, 433.11, 433.21, 433.31, 433.81, 433.91, 434.01, 434.11, 434.91, 436.x | I63 |
| Systemic embolism | 444 | I74 |
| Acute myocardial infarction | 410 | I21, I22 |
| Peripheral vascular disease | 093.0, 437.3, 440, 441, 443.1x - 443.9x, 447.1, 557.1, 557.9, V43.4 | I70, I71, I73.1, I73.8, I73.9, I77.1, I79.0, I79.2, K55.1, K55.8, K55.9, Z95.8, Z95.9 |
| Venous thromboembolism | 415.1x, 451.x, 452, 453.x | I26.9x, I80.x, I81, I82 |
| Transient ischemic attack | 435 | G45 |

Table S6.2. Codes for identification of coronary artery disease

| **Procedure** | **Code type** | **Codes** |
| --- | --- | --- |
| Coronary artery disease | ICD-9-CM Procedure | 0.66, 36.04, 36.06, 36.07, 36.03, 36.09, 36.10, 36.20, 36.30 |
|  | ICD-10-PCS Procedure | 027x, 02Cx, 210x, 211x, 212x, 213x |

**Abbreviations:** ICD-9-CM, International Classification of Diseases, Ninth Revision, Clinical Modification; ICD-10-CM, International Classification of Diseases, Tenth Revision, Clinical Modification

# Table S7. Diagnosis codes for major bleeding events

| **Outcome subtype** | **ICD-9-CM diagnosis code** | **ICD-10-CM diagnosis code** |
| --- | --- | --- |
| Intracranial hemorrhage | 430, 431, 432, 432.1, 432.9 | I60.x, I61.x, I62.1, I62.0, I62.9 |
| Gastroduodenal bleeding | 531.00, 531.20, 531.40, 531.60, 532.00, 532.20, 532.40, 532.60, 533.00, 533.20, 533.40, 533.60, 534.00, 534.20, 534.40, 534.60, 535.01, 535.11, 535.21, 535.31, 535.41, 535.51, 535.61, 537.83, 456, 456.2, 530.7, 530.82, 578, 455.2, 455.5, 455.8, 562.02, 562.03, 562.12, 562.13, 568.81, 569.3, 569.85, 578.1, 578.9 | K25.0, K25.2, K25.4, K25.6, K26.0, K26.2, K26.4, K26.6, K27.0, K27.2, K27.4, K27.6, K28.0, K28.2, K28.4, K28.6, K29.01, K29.41, K29.61, K29.21, K29.61, K29.71, K29.91, K29.51, K29.81, K31.811, I85.01, I85.11, K22.6, K22.8, K92.0, K57.01, K57.11, K57.13, K57.21, K57.31, K57.33, K57.41, K57.51, K57.53, K57.81, K57.91, K57.93, K66.1, K62.5, K55.21, K92.1, K92.2 |
| Other bleeding | 423, 459, 568.81, 719.10, 784.7, 784.8, 786.3, 593.81, 599.7, 626.6 | I31.2, R58, K66.1, M25.0, R04.0, R04.1, R04.2, R04.81, R04.89, R04.9, N28.0, R31, N92.1 |

**Abbreviations:** ICD-9, International Classification of Diseases, Ninth Revision; ICD-10, International Classification of Diseases, Tenth Revision

# Table S8. Codes for assessing CHA_2_DS_2_-VASC score

Table S8.1. Scoring algorithm for CHA_2_DS_2_-VASC Score (2)

|  | **Condition (original)** | **Condition (modified)** | **Points** |
| --- | --- | --- | --- |
| **C** | Congestive heart failure (or left ventricular systolic dysfunction) | ≥1 diagnosis for congestive heart failure | 1 |
| **H** | Hypertension: blood pressure consistently above 140/90 mmHg (or treated hypertension on medication) | ≥1 diagnosis for hypertension; OR | 1 |
|  |  | ≥1 prescription fill for antihypertensive or diuretic medications |  |
| **A_2_** | Age ≥75 years | Age ≥75 years as of the index date | 2 |
| **D** | Diabetes Mellitus | ≥1 diagnosis for diabetes mellitus; OR | 1 |
|  |  | ≥1 prescription fill for antidiabetic medications |  |
| **S_2_** | Prior Stroke or Transient Ischemic Attack or thromboembolism | ≥1 diagnosis for stroke, or TIA, or thromboembolism | 2 |
| **V** | Vascular disease (e.g. peripheral artery disease, myocardial infarction, aortic plaque) | ≥1 diagnosis for vascular disease | 1 |
| **A** | Age 65-74 years | Age 65-74 years as of the index date | 1 |
| **Sc** | Female sex | Female sex | 1 |

Table S8.2. Codes for diseases involved in CHA_2_DS_2_-VASC Score

| **Disease of interest** | **ICD-9-CM diagnosis code** | **ICD-10-CM diagnosis code** |
| --- | --- | --- |
| Congestive heart failure | 428.00x | I50.x |
| Hypertension | 401.x - 405.x | I10.x - I16.x |
| Diabetes mellitus | 250.00x | E10.x - E13.x |
| Prior stroke or transient ischemic attack or thromboembolism | 431.x - 435.x | I61.x - I66.x, G45.x |
| Vascular disease and myocardial infarction | 440.x - 449.x, 410.x, 412.x | I70.x - I79.x, I21.x, I22.x, I25.2x |

**Abbreviations:** ICD-9-CM, International Classification of Diseases, Ninth Revision, Clinical Modification; ICD-10-CM, International Classification of Diseases, Tenth Revision, Clinical Modification

Table 8.3. Codes for medications involved in CHA_2_DS_2_-VASC score

| **Medication of interest** | **ATC code** |
| --- | --- |
| Antihypertensive medications and Diuretics | C08x, C02Ax, C02Ax, C02Bx, C02Cx, C02Lx, C03Ax, C03Bx, C03Dx, C03Ex, C03Xx, C07Bx, C07Cx, C07Dx, C08Gx, C07Ax, C07Bx, C07Cx, C07Dx, C07Fx, C02DAx, C09BAx, C09DAx, C02DBx, C02DDx, C02DGx, C09BBx, C09DBx, C09AAx, C09BAx, C09BBx, C09CAx, C09Dax, C09DBx, C09XA02x, C09XA52x |
| Antidiabetic medications | A10x |

**Abbreviations:** ATC, Anatomical Therapeutic Chemical

# Table S9. Codes for assessing HAS-BLED score

Table S9.1. Scoring algorithm for HAS-BLED score

| **Abbreviation** | **Condition (original)** | **Condition (modified)** | **Points** |
| --- | --- | --- | --- |
| **H** | Hypertension | ≥1 diagnosis for hypertension | 1 |
| **A** | Abnormal renal and liver function (1 point each) | ≥1 diagnosis for renal disease; OR | 1 or 2 |
|  |  | ≥1 diagnosis for hepatic-liver disease |  |
| **S** | Stroke | ≥1 diagnosis for stroke | 2 |
| **B** | Bleeding | ≥1 diagnosis for previous bleeding | 1 |
| **L** | Labile International Normalized Ratios | N/A - Omitted | 2 |
| **E** | Elderly (>65 years) | Age >65 years as of the index date | 1 |
| **D** | Drugs or alcohol concomitantly (1 point each) | ≥1 diagnosis for ethanol abuse; OR | 1 or 2 |
|  |  | ≥1 prescription fill for non-steroidal anti-inflammatory drugs or antiplatelets |  |

Table S9.2. Codes for health conditions that are part of HAS-BLED Score

| **Disease of interest** | **ICD-9-CM diagnosis code** | **ICD-10-CM diagnosis code** |
| --- | --- | --- |
| Hypertension | 428.00x | I50.x |
| Renal disease | 250.4x, 403.xx, 404.xx, 405.01, 405.11, 405.91, 584.x, 585.x, 586.x, 588.xx, 753.0x, 753.1x, 791.0x, V45.1x, V56.xx | E10.21x, E10.29x, E10.65x, E11.21x, E11.29x, E11.65x, I12.x, I13.x, I15.0x, N17.x, N18.x, N19x, N25.x, Q60.0x, Q60.1x, Q60.2x, Q60.3x, Q60.4x, Q60.5x, Q61.x, R80.x, Z99.2x, Z91.15x, Z49.x |
| Hepatic-liver disease | 570.x, 571.xx, 572.x, 573.x, 751.62, 271.0x | K76.2x, K72.0x, K70.x, K71.7x, K73.x, K74.x, K75.4x, K75.81x, K76.0x, K76.89x, K76.9x, K72.01x, K72.1x, K72.9x, K75.0x, K75.1x, K76.6x, K76.7x, K71.x, K75.2x, K75.3x, K75.89x, K75.9x, K76.1x, K76.3x, K76.4x, K76.5x, K76.8x, K76.9x, K77.x, Q44.6x, E74.0x, E74.4x |
| Stroke | 430.x, 431.x, 432.x, 433.x, 434.x, 435.x, 436.x, 437.1x, 437.3x, 997.02 | I60.x, I61.x, I62.x, I65.x, I63.0x - I63.2x, I66.x, I63.3x-I63.9x, I67.89x, I67.81x, I67.82x, I67.89x, I67.1x, I97.81x, I97.82x, G97.3x, G45.0x-G45.2x, G45.8x, G45.9x, I67.84x |
| Previous bleeding | 423.x, 423.0x, 430.x, 431.x, 432.x, 455.2x, 455.5x, 455.8x, 456.0x, 456.20, 459.0x, 530.7x, 530.82, 531.00, 531.01, 531.20, 531.21, 531.40, 531.41, 531.60, 531.61, 532.00, 532.01, 532.20, 532.21, 532.40, 532.41, 532.60, 532.61, 533.00, 533.01, 533.20, 533.21, 533.40, 533.41, 533.60, 533.61, 534.00, 534.01, 534.20, 534.21, 534.40, 534.41, 534.60, 534.61, 535.01, 535.11, 535.21, 535.31, 535.41, 535.51, 535.61, 537.83, 562.02, 562.03, 562.12, 562.13, 568.81, 569.3x, 569.85, 578.0x, 578.1x, 578.9x, 593.81, 596.7x, 599.7x, 626.2x, 626.6x, 719.1x, 784.7x, 784.8x, 786.3x, 852.0x, 852.2x, 852.4x, 853.0x | I31.x, I31.2x, I60.x, I61.x, I62.x, K64.x, K64.x, K64.x, I85.01x, I85.11x, R58.x, K22.6x, K22.8x, K25.0x, K25.0x, K25.2x, K25.2x, K25.4x, K25.4x, K25.6x, K25.6x, K26.0x, K26.0x, K26.2x, K26.2x, K26.4x, K26.4x, K26.6x, K26.6x, K27.0x, K27.0x, K27.2x, K27.2x, K27.4x, K27.4x, K27.6x, K27.6x, K28.0x, K28.0x, K28.2x, K28.2x, K28.4x, K28.4x, K28.6x, K28.6x, K29.01x, K29.41x, K29.51x, K29.61x, K29.21x, K29.61x, K29.71x, K29.91x, K29.81x, K31.811, K57.11x, K57.13x, K57.31x, K57.33x, K66.1x, K62.5x, K55.21x, K92.0x, K92.1x, K92.2x, N28.0x, N32.89x, R31.x, N92.0x, N92.1x, M25.0x, R04.0x, R04.1x, R04.2x, R04.8x, R04.9x, S06.6Xx, S06.5Xx, S06.4Xx, S06.36xA |
| Ethanol abuse | 291.xx, 303.xx, 305.0x, 571.0x, 571.1x, 571.2x, 571.3x, 535.3x, 760.71, 980.0x | F10.x, K70.0x, K70.1x, K70.3x, K70.4x, K29.2x, P04.3x, Q86.0x  T51.0x |

**Abbreviations:** ICD-9-CM, International Classification of Diseases, Ninth Revision, Clinical Modification; ICD-10-CM, International Classification of Diseases, Tenth Revision, Clinical Modification

Table S9.3. Codes for medication classes used in HAS-BLED score

| **Medication of interest** | **ATC code** |
| --- | --- |
| Nonsteroidal anti-inflammatory agents (NSAIDs) | M01A |
| Antiplatelet | B01AC06, N02BA01, B01AC22, B01AC24, B01AC04, B01AC05 |

**Abbreviations:** ATC, Anatomical Therapeutic Chemical

# Table S10. Codes for assessing Quan-Charlson comorbidity index score

| **Comorbidities** | **ICD-9-CM diagnosis codes** (4) | **ICD-10-CM diagnosis codes** (4) | **Weight applied** (5) |
| --- | --- | --- | --- |
| Myocardial infarction | 410.x, 412.x | I21.x, I22.x, I25.2x | 0 |
| Congestive heart failure | 398.91, 402.01, 402.11, 402.91, 404.01, 404.03, 404.11, 404.13, 404.91, 404.93, 425.4x - 425.9x, 428.x | I09.9x, I11.0x, I13.0x, I13.2x, I25.5x, I42.0x, I42.5x-I42.9x, I43.x, I50.x, P29.0x | 2 |
| Peripheral vascular disease | 093.0x, 437.3x, 440.x, 441.x, 443.1x-443.9x, 447.1x 557.1x, 557.9x, V43.4x | I70.x, I71.x, I73.1x, I73.8x, I73.9x, I77.1x, I79.0x, I79.2x, K55.1x, K55.8x, K55.9x, Z95.8x, Z95.9x | 0 |
| Cerebrovascular disease | 362.34, 430.x - 438.x | G45.x, G46.x, H34.0x, I60.x - I69.x | 0 |
| Dementia | 290.x, 294.1x, 331.2x | F00.x - F03.x, F05.1, G30.x, G31.1x | 2 |
| Chronic pulmonary disease | 416.8x, 416.9x, 490.x-505.x, 506.4x, 508.1x, 508.8x | I27.8x, I27.9x, J40.x - J47.x, J60.x - J67.x, J68.4x, J70.1x, J70.3x | 1 |
| Connective tissue/rheumatic disease | 446.5x, 710.0x - 710.4x, 714.0x - 714.2x, 714.8x, 725.x | M05.x, M06.x, M31.5x, M32.x-M34.x, M35.1x, M35.3x, M36.0x | 1 |
| Peptic ulcer disease | 531.x - 534.x | K25.x-K28.x | 0 |
| Mild liver disease: | 070.22, 070.23, 070.32, 070.33, 070.44, 070.54, 070.6x, 070.9x, 570.x, 571.x, 573.3x, 573.4x, 573.8x, 573.9x, V42.7x | B18.x, K70.0x-K70.3x, K70.9x, K71.3x-K71.5x, K71.7x, K73.x, K74.x, K76.0x, K76.2x-K76.4x, K76.8x, K76.9x, Z94.4x | 2 |
| Diabetes without chronic complications/mild to moderate | 250.0x - 250.3x; 250.8x, 250.9x | E10.0x, E10.1x, E10.6x, E10.8x, E10.9x, E11.0x, E11.1x, E11.6x, E11.8x, E11.9x, E12.0x, E12.1x, E12.6x, E12.8x, E12.9x, E13.0x, E13.1x, E13.6x, E13.8x, E13.9x, E14.0x, E14.1x, E14.6x, E14.8x, E14.9x | 0 |
| Paraplegia and hemiplegia | 334.1x, 342.x, 343.x, 344.0x-344.6x, 344.9x | G04.1x, G11.4x, G80.1x, G80.2x, G81.x, G82.x, G83.0x-G83.4x, G83.9x | 2 |
| Renal disease | 403.01, 403.11, 403.91, 404.02, 404.03, 404.12, 404.13, 404.92, 404.93, 582.0x - 583.7x, 585.x, 586.x, 588.0x, V42.0x, V45.1x, V56.x | I12.0x, I13.1x, N03.2x-N03.7x, N05.2x-N05.7x, N18.x, N19.x, N25.0x, Z49.0x-Z49.2x, Z94.0x, Z99.2x | 1 |
| Diabetes with chronic complications | 250.4x - 250.7x | E10.2x-E10.5x, E10.7x, E11.2x-E11.5x, E11.7x, E12.2x-E12.5x, E12.7x, E13.2x-E13.5x, E13.7x, E14.2x-E14.5x, E14.7x | 1 |
| Any malignancy, including lymphoma and leukemia, except malignant neoplasm of skin | 140.x - 172.x, 174.x - 195.8x, 200.x - 208.x, 238.6x | C00.x-C26.x, C30.x-C34.x, C37.x-C41.x, C43.x, C45.x-C58.x, C60.x-C76.x, C81.x-C85.x, C88.x, C90.x-C97.x | 2 |
| Moderate or severe liver disease | 456.0x-456.2x, 572.2x-572.8x | I85.0x, I85.9x, I86.4x, I98.2x, K70.4x, K71.1x, K72.1x, K72.9x, K76.5x, K76.6x, K76.7x | 4 |
| Metastatic solid tumor | 196.x - 199.x | C77.x-C80.x | 6 |
| AIDS/HIV | 042.x, 043.x, 044.x | B20.x-B22.x, B24.x | 4 |

**Abbreviations:** AIDS, acquired immunodeficiency syndrome; ICD-9-CM, International Classification of Diseases, Ninth Revision, Clinical Modification; ICD-10-CM, International Classification of Diseases, Tenth Revision, Clinical Modification; HIV, human immunodeficiency virus

# Table S11. Codes for identifying baseline characteristics

| **Condition** | **ICD-9-CM diagnosis codes** | **ICD-10-CM diagnosis codes** |
| --- | --- | --- |
| Anemia | 280.x - 285.x | D50.x - D64.x |
| Asthma | 493.00x | J45.x |
| Cancers | 140.x - 208.x | C00.x - C26.x, C30.x - C34.x, C37.x - C41.x, C43.x, C4A.x, C44.x - C58.x, C60.x - C96.x |
| Cerebrovascular disease | 430, 431, 432, 433, 434, 435, 436, 437, 438, 362.34, 997.02 | G45, G46, I60, I61, I62, I63, I64, I65, I66, I67, I68, I69 H34.0, G97.3 I97.81, I97.82 |
| Congestive heart failure | 428.00x | I50.x |
| Myocardial infarction | 410.00x, 412.00x | I21.x, I22.x, I25.2x |
| Peripheral vascular disease | 093.0x, 437.3x, 440.x, 441.x, 443.1x-443.9x, 447.1x, 557.1x, 557.9x, V43.4x | I70.x, I71.x, I73.1x, I73.8x, I73.9x, I77.1x, I79.0x, I79.2x, K55.1x, K55.8x, K55.9x, Z95.8x, Z95.9x |
| Chronic obstructive pulmonary disease | 491.x, 492.x, 493.2x, 496.x | J41.x, J42.x, J43.x, J44.x |
| Diabetes mellitus | 250.00x | E10.x-E13.x |
| Ulcer (gastric, duodenal, gastrojejunal, esophageal, or peptic ulcer site specified) | 530.2x, 531.x-534.x | K22.1x, K25.x-K28.x |
| Hypertension | 401.x - 405.x | I10.x - I16.x |
| Hyperlipidemia | 272.0x, 272.1x, 272.2x, 272.4x | E78.0x, E78.1x, E78.2x, E78.4x, E78.5x |
| Liver disease | 570, 571, 572, 573, 271.0, 571.8, 751.62 | K70, K71, K72, K73, K74, K75, K76, K77, B15, B16, B17, B18, B19, C22, K76.2, K72.0, K71.7, K75.4, K76.0, K76.9, K72.1, K72.9, K75.0, K75.1, K76.6, K76.7, K75.2, K75.3, K75.9, K76.1, K76.3, K76.4, K76.5, K76.8, K76.9, Q44.6, E74.0, E74.4, Z94.4, K75.81, K76.89, K72.01, K75.89 |
| Prior bleeding |  |  |
| Gastrointestinal bleeding | 578, 456.0, 456.2, 531.0, 531.2, 531.4, 531.6, 532.0, 532.2, 532.4, 532.6, 533.0, 533.2, 533.4, 533.6, 534.0, 534.2, 534.4, 534.6, 530.7, 455.2, 455.5, 455.8, 569.3, 578.1, 578.9, 535.01, 535.11, 535.21, 535.31, 535.41, 535.51, 535.61, 535.71, 537.83, 537.84, 530.21, 530.82, 562.02, 562.03, 562.12, 562.13, 568.81, 569.85 | K25.0, K25.2, K25.4, K25.6, K26.0, K26.2, K26.4, K26.6, K27.0, K27.2, K27.4, K27.6, K28.0, K28.2, K28.4, K28.6, K22.6, K22.8, K92.0, K66.1, K62.5, K92.0, K92.1, K92.2, K29.01, K29.41, K29.61, K29.21, K29.61, K29.71, K29.91, K29.51, K29.81, I85.01, I85.11, K57.01, K57.11, K57.13, K57.21, K57.31, K57.33, K57.41, K57.51, K57.53, K57.81, K57.91, K57.93, K55.21, K31.82, K22.11, K31.811 |
| Intracerebral hemorrhage | 430, 431, 432, 432.1, 432.9 | I60, I61, I62, I62.0, I62.1, I62.9 |
| Other bleeding | 626.6, 784.7, 784.8, 423.0, 719.1, 786.3, 363.6, 459.0, 599.7, 593.81, 568.81, 376.32, 377.42, 379.23, 729.92 | R31, R58, D62, N02, N92.1, R04.0, R04.1, I31.2, K66.1, M25.0, R04.2, R04.9, H31.3, H43.1, J94.2, H11.3, H35.6, N95.0, N28.0, R04.81, R04.89, M79.81 |
| Thyroid disease | 240.x - 246.x | E00.x - E07.x |
| Venous thromboembolism | 415.1x, 451.x, 452, 453.x | I26.9x, I80.x, I81, I82 |

**Abbreviations:** ICD-9-CM, International Classification of Diseases, Ninth Revision, Clinical Modification; ICD-10-CM, International Classification of Diseases, Tenth Revision, Clinical Modification

# Table S12. ATC codes for baseline medication use

| **Drug class/subclass** | **ATC code start with** |
| --- | --- |
|  |  |
| Antianxiety agents | N05Bx |
| Antiarrhythmic agents | C01Bx, C01EB10 |
| Anti-depressants | N06Ax |
| Antiplatelets | B01ACx |
| Anti-hyperlipidemics | C10x |
| ACEIs / ARBs | C09x |
| Beta-blockers | C07x |
| Calcium channel blockers | C08x |
| Diuretics | C03x |
| Anti-hypertensives | C02x |
| Insulin | A10Ax |
| Anti-diabetics | A10Bx |
| NSAIDs | M01Ax |
| Proton pump inhibitors | A02BCx |

**Abbreviations:** ACEI, angiotensin converting enzyme inhibitors; ARB, angiotensin II receptor blockers; ATC, Anatomical Therapeutic Chemical; NSAIDs, non-steroidal anti-inflammatory drugs

# Table S13. Dosage distribution in the study population using DOACs

| Medication | Patients number | (%) |
| --- | --- | --- |
| **Warfarin** | **202** | **(20.0)** |
| **DOACs** | **809** | **(80.0)** |
| Dabigatran | 155 | (19.2) |
| 75 mg BID | 2 | (1.3) |
| 110 mg BID | 135 | (87.1) |
| 150 mg BID | 8 | (5.2) |
| Other | 10 | (6.5) |
| Rivaroxaban | 257 | (31.8) |
| 10 mg QD | 149 | (58.0) |
| 15 mg QD | 97 | (37.7) |
| 20 mg QD | 1 | (0.4) |
| Other | 10 | (3.9) |
| Apixaban | 255 | (31.5) |
| 2.5 mg BID | 217 | (85.1) |
| 5 mg BID | 28 | (11.0) |
| Other | 10 | (3.9) |
| Edoxaban | 142 | (17.6) |
| 30 mg QD | 131 | (92.3) |
| 60 mg QD | 4 | (2.8) |
| Other | 7 | (5.0) |
| Total number | 1011 | (100) |

**Abbreviations:** BID, twice daily; QD, once daily; DOACs, direct oral anticoagulants

# Table S14. Distribution of TTR in the study population using warfarin

| TTR | Patients number | (%) |
| --- | --- | --- |
| 90-100% | 7 | (3.5) |
| 80-89% | 6 | (3.0) |
| 70-79% | 10 | (5.0) |
| 60-69% | 11 | (5.4) |
| 50-59% | 23 | (11.4) |
| 40-49% | 10 | (5.0) |
| 30-39% | 11 | (5.4) |
| 20-29% | 16 | (7.9) |
| 10-19% | 6 | (3.0) |
| 0-9% | 30 | (14.9) |
| Unknown | 72 | (35.6) |
| **Total number** | **202** | **(100)** |
|  |  |  |

The mean TTR without the unknown (n=130) was 39.5%. After incorporating IPTW, the mean TTR was 48.2%. The result of the sensitivity analysis that only included warfarin users with TTR ≥70% (n=23) is shown in the supplemental eTable 18. Furthermore, only 11.4% (23/202) of warfarin users in our study population could achieve the goal of TTR ≥70%.

**Abbreviations:** TTR, time in therapeutic range

# Table S15. Unweighted incidence rates and hazard ratios of outcomes

| Outcome | DOACs group (n=809) | | |  | Warfarin group (n=202) | | | HR (95% CI) |
| --- | --- | --- | --- | --- | --- | --- | --- | --- |
|  | Events | PY | Rate^a^ (95%CI) |  | Events | PY | Rate^a^ (95%CI) |  |
| Stroke/SE | 7 | 339 | 2.07 (0.99-4.35) |  | 5 | 114 | 4.37 (1.82-10.49) | 0.43 (0.13-1.43) |
| Major bleeding | 15 | 337 | 4.45 (2.68-7.38) |  | 6 | 112 | 5.34 (2.40-11.89) | 0.75 (0.29-1.94) |
| Any ischemia | 27 | 330 | 8.20 (5.62-11.95) |  | 18 | 108 | 16.70 (10.52-26.51) | 0.44 (0.24-0.82) |
| Any bleeding | 90 | 315 | 28.53 (23.21-35.08) |  | 29 | 91 | 31.95 (22.20-45.97) | 0.81 (0.53-1.24) |

^a^ Incidence rate, per 100 person-years

**Abbreviations**: DOACs, direct oral anticoagulants; PY person-years; SE systemic embolism

# Table S16. Reasons for censorship of outcomes in the study population

| Censorship, % | Dabigatran  (N = 155) | Rivaroxaban  (N = 257) | Apixaban  (N = 255) | Edoxaban  (N = 142) | Warfarin  (N = 202) |
| --- | --- | --- | --- | --- | --- |
| Stroke or systemic embolism (stroke/SE) | | | | | |
| Study End | 0.0 | 3.9 | 8.2 | 5.6 | 3.0 |
| Outcome | 1.3 | 1.2 | 0.0 | 1.4 | 2.5 |
| Death | 2.6 | 3.9 | 3.5 | 2.8 | 2.0 |
| Discontinuation | 6.5 | 9.0 | 6.7 | 9.2 | 8.9 |
| Switching | 9.0 | 5.8 | 2.8 | 3.5 | 7.9 |
| Recovery of renal function | 50.3 | 38.1 | 32.6 | 35.9 | 24.3 |
| Unknown renal function | 29.7 | 37.0 | 44.7 | 39.4 | 48.0 |
| Disenrollment^†^ | 0.7 | 1.2 | 1.6 | 2.1 | 3.5 |
| Major Bleeding |  |  |  |  |  |
| Study End | 0.0 | 3.9 | 7.8 | 5.6 | 3.0 |
| Outcome | 2.6 | 2.3 | 0.8 | 2.1 | 3.0 |
| Death | 2.6 | 3.9 | 3.5 | 2.8 | 2.5 |
| Discontinuation | 6.5 | 9.0 | 6.7 | 8.5 | 8.9 |
| Switching | 8.4 | 5.5 | 2.8 | 3.5 | 8.4 |
| Recovery of renal function | 51.0 | 38.1 | 32.2 | 35.2 | 24.3 |
| Unknown renal function | 28.4 | 36.2 | 44.7 | 40.1 | 46.5 |
| Disenrollment^†^ | 0.7 | 1.2 | 1.6 | 2.1 | 3.5 |
| Any Ischemia |  |  |  |  |  |
| Study End | 0.0 | 3.9 | 8.2 | 5.6 | 3.0 |
| Outcome | 4.5 | 3.9 | 2.4 | 2.8 | 8.9 |
| Death | 2.6 | 3.9 | 2.8 | 2.8 | 2.0 |
| Discontinuation | 5.8 | 9.0 | 6.7 | 9.2 | 8.4 |
| Switching | 9.0 | 5.5 | 2.8 | 3.5 | 6.4 |
| Recovery of renal function | 49.7 | 37.4 | 31.8 | 35.2 | 23.8 |
| Unknown renal function | 27.7 | 35.8 | 44.7 | 38.7 | 44.6 |
| Disenrollment^†^ | 0.7 | 0.8 | 0.8 | 2.1 | 3.0 |
| Any Bleeding |  |  |  |  |  |
| Study End | 0.0 | 3.5 | 7.1 | 5.6 | 1.5 |
| Outcome | 13.6 | 13.6 | 7.8 | 9.9 | 14.4 |
| Death | 1.3 | 3.5 | 2.8 | 2.8 | 1.0 |
| Discontinuation | 5.2 | 7.4 | 6.3 | 7.0 | 7.4 |
| Switching | 7.1 | 3.9 | 2.8 | 2.8 | 7.4 |
| Recovery of renal function | 46.5 | 33.9 | 29.8 | 32.4 | 22.8 |
| Unknown renal function | 25.8 | 33.9 | 42.0 | 37.3 | 42.6 |
| Disenrollment^†^ | 0.7 | 0.4 | 1.6 | 2.1 | 3.0 |

^†^ Including the incidence of valve surgery, kidney transplantation, or mitral stenosis diagnosis after index date

# Table S17. Results of sensitivity analyses

| Outcome | DOACs group | | |  | Warfarin group | | | Adjusted^b^  HR (95% CI) | P_int_ |
| --- | --- | --- | --- | --- | --- | --- | --- | --- | --- |
|  | Events/ patients | PY | Rate**^a^** (95%CI) |  | Events/ patients | PY | Rate**^a^** (95%CI) |  |  |
| **Excluded unreasonable dosage of DOACs** | | | | | | | | | |
| Stroke/SE | 4/387 | 164 | 2.18 (0.77-6.13) |  | 17/508 | 259 | 6.54 (4.06-10.53) | 0.29 (0.07-1.11) | 1.00 |
| Major bleeding | 7/387 | 164 | 4.15 (1.96-8.79) |  | 7/508 | 257 | 2.74 (1.31-5.74) | 0.92 (0.29-2.99) | 0.93 |
| Any ischemia | 12/387 | 160 | 7.42 (4.20-13.11) |  | 35/508 | 248 | 14.18 (10.18-19.74) | 0.40 (0.20-0.81)* | 0.92 |
| Any bleeding | 43/387 | 154 | 28.20 (20.94-37.98) |  | 65/508 | 202 | 32.31 (25.33-41.20) | 0.68 (0.45-1.04) | 0.77 |
| **Only included warfarin users with TTR ≥70%** | | | | | | | | | |
| Stroke/SE | 5/503 | 208 | 2.25 (0.91-5.56) |  | 1/68 | 45 | 2.26 (0.33-15.60) | 0.91 (0.07-12.81) | 0.43 |
| Major bleeding | 9/503 | 208 | 4.33 (2.25-8.33) |  | 1/68 | 45 | 2.26 (0.33-15.60) | 0.83 (0.09-7.88) | 0.89 |
| Any ischemia | 17/503 | 202 | 8.27 (5.12-13.34) |  | 6/68 | 45 | 13.81 (6.28-30.39) | 0.30 (0.10-0.87)* | 0.60 |
| Any bleeding | 55/503 | 194 | 28.38 (21.80-36.95) |  | 4/68 | 44 | 10.08 (3.98-25.53) | 1.89 (0.69-5.19) | 0.09 |
| **Utilized the MDRD formula to estimate GFR** | | | | | | | | | |
| Stroke/SE | 3/268 | 96 | 2.73 (0.82-9.14) |  | 30/292 | 119 | 24.90 (17.37-35.68) | 0.16 (0.04-0.73)* | 0.53 |
| Major bleeding | 4/268 | 95 | 4.67 (1.85-11.81) |  | 6/292 | 117 | 5.42 (2.49-11.83) | 1.40 (0.34-5.82) | 0.70 |
| Any ischemia | 8/268 | 95 | 8.25 (4.10-16.61) |  | 35/292 | 118 | 29.65 (21.28-41.31) | 0.34 (0.14-0.84)* | 0.71 |
| Any bleeding | 26/268 | 91 | 28.29 (19.23-41.64) |  | 50/292 | 103 | 49.08 (37.24-64.67) | 0.51 (0.31-0.85)* | 0.25 |
| **Restricted follow-up period to only 1 year** | | | | | | | | | |
| Stroke/SE | 4/503 | 187 | 2.18 (0.83-5.75) |  | 17/508 | 196 | 8.62 (5.35-13.89) | 0.23 (0.06-0.83)* | 0.80 |
| Major bleeding | 8/503 | 186 | 4.47 (2.27-8.82) |  | 6/508 | 196 | 3.22 (1.48-7.02) | 1.09 (0.35-3.42) | 0.90 |
| Any ischemia | 15/503 | 185 | 8.29 (5.02-13.68) |  | 31/508 | 192 | 16.13 (11.34-22.94) | 0.41 (0.21-0.80)* | 0.96 |
| Any bleeding | 53/503 | 176 | 29.92 (22.85-39.18) |  | 63/508 | 168 | 37.48 (29.29-47.96) | 0.74 (0.50-1.09) | 1.00 |
| **Excluded patients with CrCl <30 mL/min only once** | | | | | | | | | |
| Stroke/SE | 3/309 | 165 | 1.83 (0.59-5.66) |  | 5/371 | 235 | 2.29 (0.98-5.33) | 0.52 (0.12-2.37) | 0.55 |
| Major bleeding | 8/309 | 164 | 5.14 (2.62-10.09) |  | 6/371 | 234 | 2.70 (1.23-5.89) | 0.90 (0.27-3.01) | 0.91 |
| Any ischemia | 14/309 | 160 | 8.73 (5.17-14.76) |  | 21/371 | 224 | 9.28 (6.04-14.26) | 0.78 (0.38-1.60) | 0.21 |
| Any bleeding | 42/309 | 153 | 27.44 (20.27-37.14) |  | 55/371 | 178 | 30.65 (23.51-39.97) | 0.80 (0.51-1.25) | 0.80 |

^a^ Incidence rate, per 100 person-years

^b^ Weighted with inverse probability of treatment weighting (IPTW) and adjusted for age, sex, chronic obstructive pulmonary disease, gastrointestinal ulcer, diuretics, antidiabetics, CHA_2_DS_2_-VASc score, HAS-BLED score, smoking status, prior bleeding, cerebrovascular disease, myocardial infarction, peripheral vascular disease, venous thromboembolism, antiplatelets, non-steroidal anti-inflammatory drugs

**Abbreviations**: CrCl, creatinine clearance according to the Cockcroft-Gault equation; DOACs, direct oral anticoagulants; MDRD, Modification of Diet in Renal Disease; PY, person-years; SE, systemic embolism

**TTR and outcomes**

In the sensitivity analysis of only including warfarin users with TTR ≥ 70%, we found the borderline significance of DOACs to increase the risk of any bleeding events compared with warfarin. This indicates that the comparative safety between DOACs and warfarin in this population may be linked to the TTR of warfarin users. Current clinical guidelines suggest that well-managed warfarin therapy (ideally TTR ≥70%) is an effective and relatively safe drug. A previous study showed that TTR negatively correlates with thromboembolic and major bleeding rates. Unfortunately, the majority of patients in our analysis population, and even in other studies, did not attain an optimal TTR. Some studies did not reveal the TTR of their warfarin population, which restricts the interpretation and applicability of the results. Furthermore, AKD might be the sole predictor of poor TTR. These outcomes have implications for warfarin users with AKD, who must tightly control their INR or switch warfarin to DOACs to mitigate the risk of bleeding.


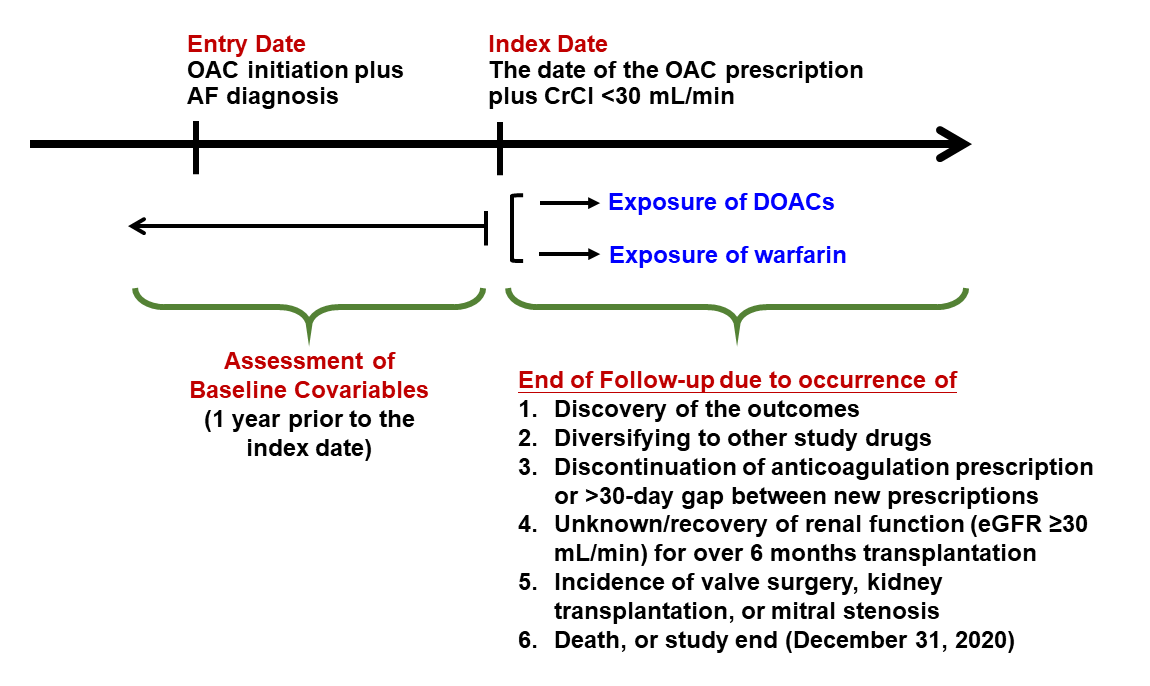


# Figure S1. Study design and follow-up scheme

**Abbreviations:** AF, atrial fibrillation; CrCl, creatinine clearance; DOACs, direct oral anticoagulants; OAC, oral anticoagulant


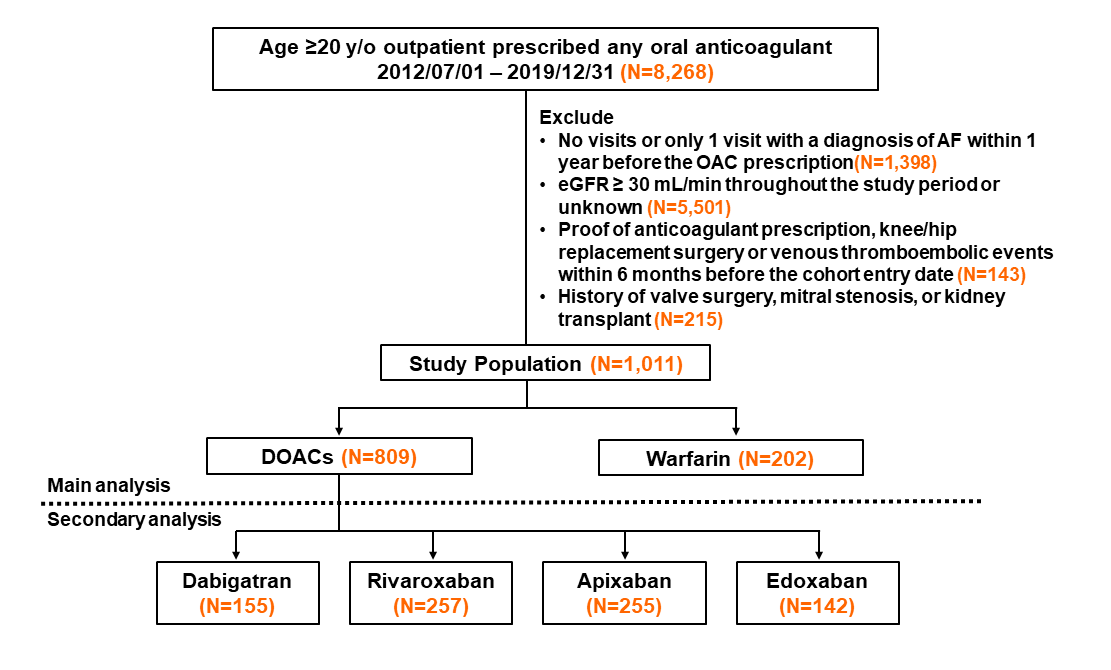


# Figure S2. Flow chart for the enrollment of the study population

**Abbreviations:** y/o, years old; AF, atrial fibrillation; CrCl, creatinine clearance; DOACs, direct oral anticoagulants; OAC, oral anticoagulant

| **A. Stroke and systemic embolism** | **B. Major bleeding** |
| --- | --- |
| **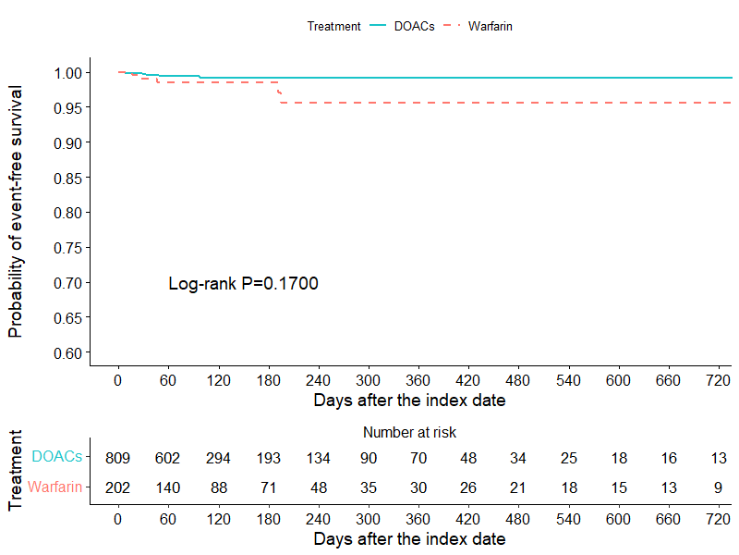** | **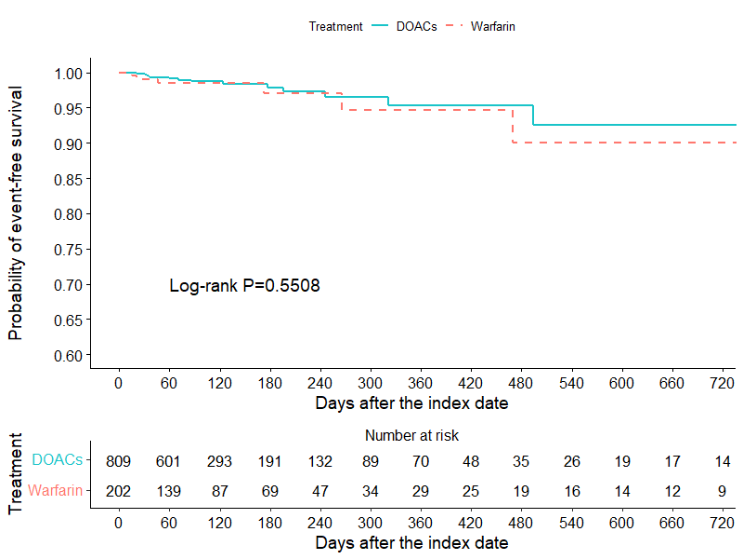** |
| **C. Any ischemia** | **D. Any bleeding** |
| **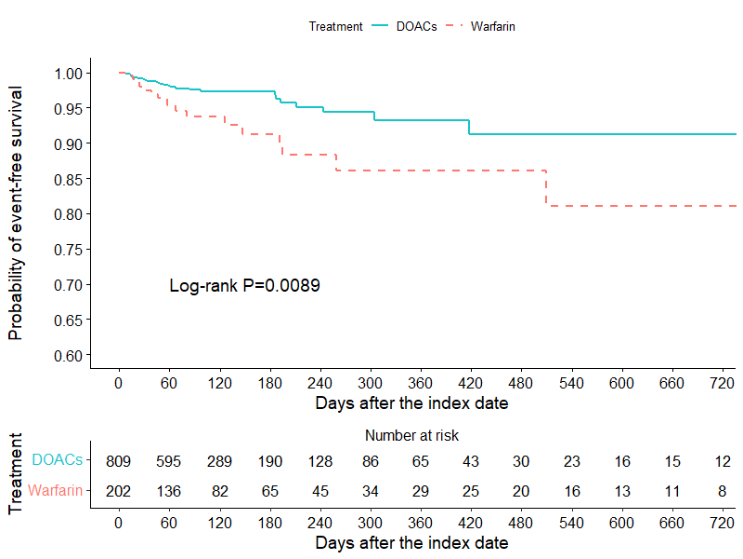** | **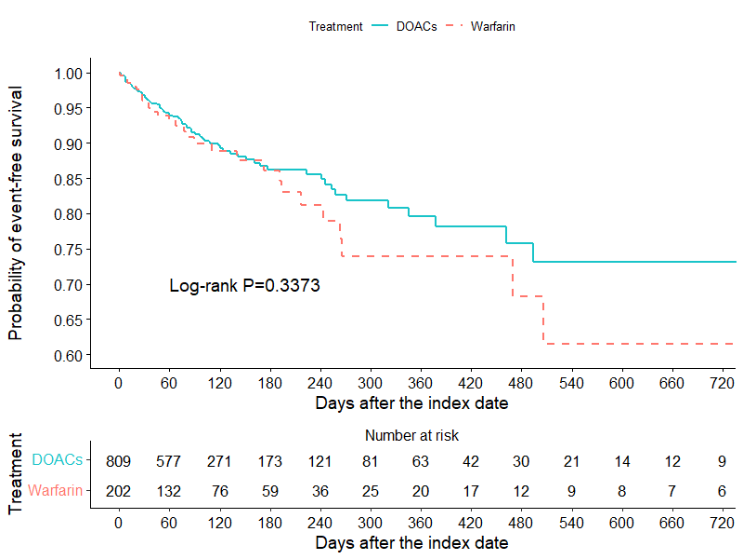** |

# Figure S3. Unweighted Kaplan-Meier survival curves in patients with atrial fibrillation and advanced kidney disease

(A) Stroke and systemic embolism. (B) Major bleeding. (C) Any ischemia. (D) Any bleeding

**Abbreviations:** DOACs, direct oral anticoagulants


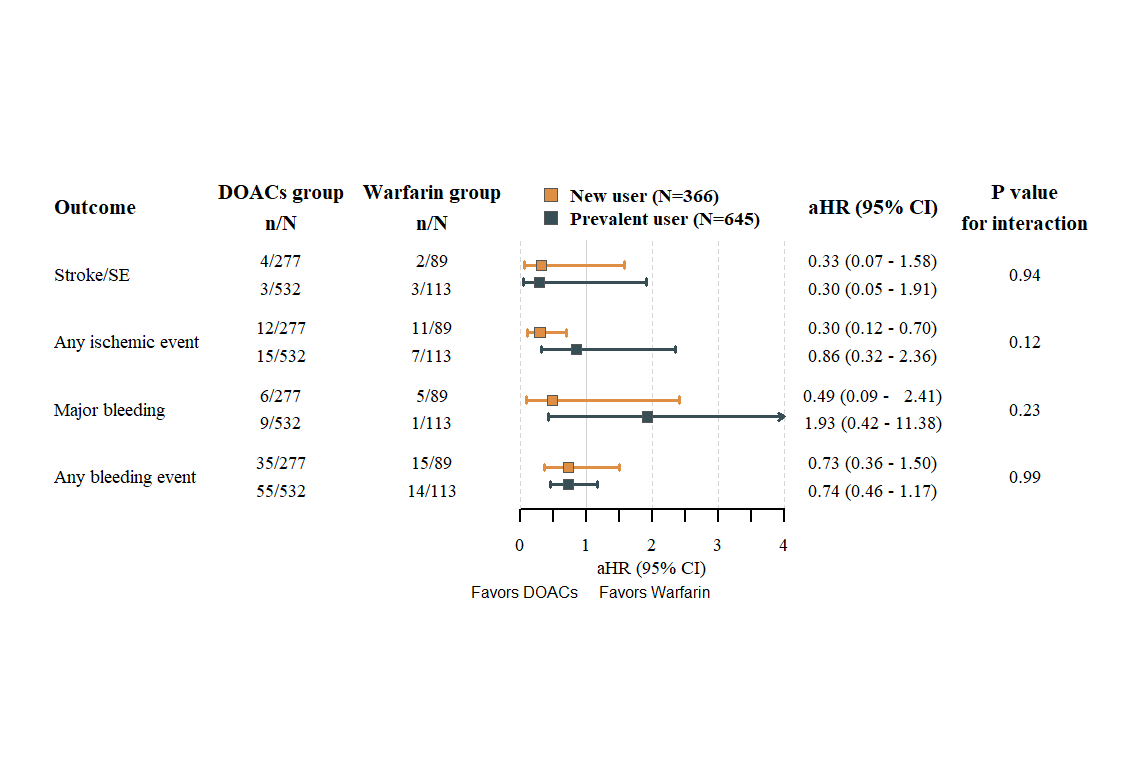


# Figure S4. Subgroup analysis stratified by the new and prevalent user group and interaction P value

# References

1. Cunningham A, Stein CM, Chung CP, Daugherty JR, Smalley WE, Ray WA: An automated database case definition for serious bleeding related to oral anticoagulant use. Pharmacoepidemiol Drug Saf, 20: 560-566, 2011
2. Lip GY, Nieuwlaat R, Pisters R, Lane DA, Crijns HJ: Refining clinical risk stratification for predicting stroke and thromboembolism in atrial fibrillation using a novel risk factor-based approach: the euro heart survey on atrial fibrillation. Chest, 137: 263-272, 2010
3. Pisters R, Lane DA, Nieuwlaat R, de Vos CB, Crijns HJ, Lip GY: A novel user-friendly score (HAS-BLED) to assess 1-year risk of major bleeding in patients with atrial fibrillation: the Euro Heart Survey. Chest, 138: 1093-1100, 2010
4. Quan H, Sundararajan V, Halfon P, Fong A, Burnand B, Luthi JC, Saunders LD, Beck CA, Feasby TE, Ghali WA: Coding algorithms for defining comorbidities in ICD-9-CM and ICD-10 administrative data. Med Care, 43: 1130-1139, 2005
5. Quan H, Li B, Couris CM, Fushimi K, Graham P, Hider P, Januel JM, Sundararajan V: Updating and validating the Charlson comorbidity index and score for risk adjustment in hospital discharge abstracts using data from 6 countries. Am J Epidemiol, 173: 676-682, 2011

Effectiveness and Safety of Direct Oral Anticoagulants versus Warfarin in Patients with Atrial Fibrillation and Advanced Kidney Disease
